# Daytime Ventilatory and Blood Acid–Base Responses During Ascent to 3500 m Are Larger and More Rapid in Females Than Males

**DOI:** 10.1002/cph4.70279

**Published:** 2026-10-24

**Authors:** Nicole V. Bushfield, Benjamin W. L. MacKenzie, Nicole A. Johnson, Rodion Isakovich, Jamie L. Keess, Nicholas D. J. Strzalkowski, Gustavo Zubieta‐Calleja, Richard J. A. Wilson, Trevor A. Day

**Affiliations:** ^1^ Department of Biology, Faculty of Science and Technology Mount Royal University Calgary Alberta Canada; ^2^ Department of Emergency Medicine, Cumming School of Medicine University of Calgary Calgary Alberta Canada; ^3^ Mineral Springs Hospital Banff Alberta Canada; ^4^ High Altitude Pulmonary and Pathology Institute (HAPPI‐IPPA) La Paz Bolivia; ^5^ Department of Physiology and Pharmacology, Cumming School of Medicine University of Calgary Calgary Alberta Canada

**Keywords:** acid–base homeostasis, high altitude, renal compensation, sex differences, ventilatory acclimatization

## Abstract

With ascent to high altitude (HA), increased ventilation reduces the pressure of P_a_CO_2_ (i.e., hypocapnia), resulting in respiratory alkalosis. A subsequent renal compensation is mediated through reductions in [HCO_3_^−^]_a_, partly‐correcting pH_a_. High inter‐individual variability exists in ventilation, blood oxygenation, and acid–base responses with ascent to HA, but potential sex differences have not been well‐characterized. We recruited 38 lowland participants (20 M; 18 F) before and after rapid ascent to and residence at 3500 m over 10 days in La Paz, Bolivia. We performed radial arterial blood draws to assess blood oxygenation and acid–base variables before (1100 m), and following ascent to 3500 m both early HA (E‐HA; D2/3) and late HA (L‐HA; D9/10). With E‐HA, all participants had higher calculated *V̇*_A_, and lower P_a_O_2_ and S_a_O_2_ (*p* < 0.0001), with subsequent increases in $\dot{V}$_A_, [Hb] and C_a_O_2_ L‐HA (*p* = 0.0056; *p* = 0.0003; *p* = 0.0013). Although females had higher $\dot{V}$_A_ and P_a_O_2_ than males E‐HA (*p* < 0.0001; *p* = 0.0143), males had higher [Hb] and C_a_O_2_ at 1100 m, at both E‐HA (*p* = 0.0008; *p* = 0.0085) and L‐HA (*p* = 0.0007; *p* = 0.0045). E‐HA, all participants had lower P_a_CO_2_ and [HCO_3_^−^]_a_, and higher pH_a_ than 1100 m (*p* < 0.0001; *p* = 0.0004, *p* < 0.0001), suggesting respiratory alkalosis and partial renal compensation. L‐HA, P_a_CO_2_ and [HCO_3_^−^]_a_ were further reduced (*p* = 0.0062; *p* = 0.0202), with pH_a_ only partly‐compensated (*p* = 0.4306). E‐HA, P_a_CO_2_, [HCO_3_^−^]_a_ and pH_a_ were significantly lower in females (*p* < 0.0001; *p* < 0.0001; *p* = 0.0457), suggesting more complete renal compensation. However, these sex‐differences were not apparent at L‐HA (*p* > 0.5341). This is the first clear demonstration of differential ventilatory, oxygenation and acid–base compensation between sexes with rapid ascent to HA.

## Graphical abstract

We obtained arterial blood gas samples in 38 healthy lowlander participants (18 female; 20 male) before (D0) and undergoing rapid (D2/3) ascent to and resident at (D9/10) 3500 m. Females had a larger magnitude and more rapid time‐course of ventilatory and renally‐mediated blood acid–base acclimatization with rapid ascent. Males normalize to female values within 1 week.

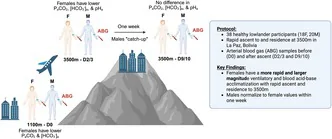

## 1 Introduction

With ascent to high altitude, there is a reduction in atmospheric pressure that lowers the partial pressure of inspired oxygen, leading to a decrease in the pressure of arterial oxygen (P_a_O_2_; i.e., hypoxemia). A decreased P_a_O_2_ leads to a decrease in arterial oxygen saturation (S_a_O_2_) and oxygen content (C_a_O_2_). This systemic oxygen deficiency activates a series of physiological responses that protect blood oxygenation and acid–base homeostasis, mediated through an integration between respiratory and renal acclimatization. These integrated responses are critical in the maintenance of blood oxygenation and acid–base homeostasis (Krapf et al. 1991; Zouboules et al. 2018; Bird et al. 2021; Johnson, Strzalkowski, et al. 2025).

Acutely, hypoxemia elicits the hypoxic ventilatory response (HVR) via activation of the peripheral chemoreceptors (i.e., carotid bodies). The increase in carotid body activation synapses with the brainstem respiratory centers, eliciting a feedback control‐mediated increase in alveolar ventilation (Powell et al. 1998; Teppema and Dahan 2010; Ainslie et al. 2013). An increase in alveolar ventilation in turn protects blood oxygen levels. However, increased ventilation has blood acid–base implications, as it results in a decrease in the partial pressure of arterial carbon dioxide (P_a_CO_2_), causing hypocapnia and respiratory alkalosis (pH_a_ > 7.4; Krapf et al. 1991; Mallet et al. 2023). This respiratory alkalosis triggers the kidneys to mount a compensatory metabolic acidosis to return blood pH to its baseline values (pHa ~7.4; Krapf et al. 1991; Zouboules et al. 2018; Bird et al. 2021). This renally mediated metabolic acidosis occurs is through a reduced hydrogen ion secretion (i.e., retention) and increased bicarbonate ion excretion (i.e., reduced reabsorption) in the renal proximal convoluted tubules and collecting duct intercalated cells (Krapf et al. 1991; Roy et al. 2015). Depending upon the magnitude of the acid–base disturbance, this compensatory metabolic acidosis facilitates the partial correction of pH_a_ values, protecting acid–base homeostasis (Krapf et al. 1991).

Ventilatory acclimatization manifests as increased sensitivity of the HVR response (Teppema and Dahan 2010), mediated by carotid body hypertrophy and hyperplasia (e.g., Powell 2007; Sato et al. 1992). Increased carotid body sensitivity to hypoxia is observed within the first 3 days of sustained hypoxia (Wang et al. 1985; Vizek et al. 1987). Additionally, the central nervous system displays plasticity in the form of increased sensitivity in the integration of afferent signaling from the carotid bodies (Moya et al. 2020). The overarching response is an increase in ventilation for a given hypoxic stimulus (Dempsey et al. 2014; Powell 2007; Moya et al. 2020; Leacy et al. 2021). Ventilatory acclimatization protects against the initial central and peripheral blunting effect of hypocapnia, initiated by the acute HVR with ascent (i.e., poikilocapnic hypoxia; Daristotle et al. 1987; Lahiri and DeLaney 1975; Steinback and Poulin 2007). Therefore, ventilatory acclimatization allows the HVR to further protect oxygenation, but further intensifies the respiratory alkalosis (Dempsey and Forster 1982; Robbins 2007). The renal response to respiratory alkalosis also affects and likely contributes to ventilatory acclimatization. For example, the reproduced [bicarbonate] in the central compartment increases the sensitivity of the central chemoreceptors to changes in P_a_CO_2_ (Dempsey et al. 2014; Forster et al. 1975; Fan et al. 2010). Additionally, the compensatory relative metabolic acidosis withdraws the blunting of the peripheral chemoreceptors originating from the initial hypocapnia and respiratory alkalosis (Dempsey et al. 2014).

Sex differences exist in these integrated respiratory‐renal responses to altitude. Females exhibit greater baseline ventilation and lower P_a_CO_2_ than males at both sea level and high altitude (e.g., M. P. Fitzgerald 1913; Fitzgerald and Haldane 1905; Loeppky et al. 2001). This sex difference is likely due to progesterone mediated stimulation of ventilation, which is particularly pronounced in the luteal phase of the menstrual cycle (Fitzgerald and Haldane 1905; M. P. Fitzgerald 1913). In females, lower P_a_CO_2_ contributes to a compensated respiratory alkalosis, which persists during altitude exposure and is further enhanced by increased progesterone levels (Loeppky et al. 2001). However, despite these differences in baseline physiology Loeppky et al. (2001) showed that women have a similar increase in ventilation in response to high altitude as men regardless of progesterone levels. This suggests that ventilatory responsiveness to acute altitude exposure is comparable between the sexes, despite differences in baseline ventilatory parameters.

There is significant variability in both the time course and magnitude of the integrated respiratory‐renal responses to high altitude. Previous research investigated sex differences in the respiratory chemoreflex and identified increased sensitivity in men (Lombardi et al. 2013; Caravita et al. 2015; Li et al. 2022). However, inconsistencies are noted in reports of sex differences in the HVR. Several studies found the HVR to be similar between sexes (Guenette et al. 2004; MacNutt et al. 2012; Rispen et al. 2017; Oeung et al. 2023). Conversely, other studies have shown a higher HVR sensitivity in males (White et al. 1983; Goldberg et al. 2017), while others have identified a larger HVR in females (Aitken, Franklin, and Cameron 1986). Additionally, Bird et al. (2023) revealed a greater sensitivity in the chemoreflex feedback loop gain, a measure of chemoreflex sensitivity, following ventilatory disturbances in males compared to females. However, Patrician et al. (2024) highlight that it is unknown if sex differences in the respiratory chemoreflex sensitivity persist following acclimatization and prolonged stay at altitude. Regardless, during sustained exposure to hypoxia, a larger steady‐state HVR is associated with enhanced hypocapnia and larger magnitude respiratory alkalosis and thus, a larger hypocapnic stimulus for renal compensation.

Ninety percent of bicarbonate reabsorption occurs in the proximal convoluted tubules (Roy et al. 2015), where previous literature has identified sexual dimorphism. Harris et al. (2019) found the proximal convoluted tubules account for ~40% of cortical volume in female mice compared to ~60% in males, which correlated with an increase in collecting duct volume in females. Veiras et al. (2017) identified differential patterns between the sexes in the transportation and handling of salts and differences in basal ammonia metabolism. The differences in tubular mechanisms resulted in female mice being associated with decreased bicarbonate reabsorption compared to their male counterparts in response to an overnight fast (Veiras et al. 2017). Thus, differences in renal morphology and/or cellular mechanisms may exist for differential renal compensation in response to sustained respiratory alkalosis between the sexes in mammals, but how these differences translate to humans remains unclear, warranting further study.

Here we address these gaps in our understanding of sex differences in ventilatory and acid–base responses at high altitude. We aimed to evaluate the impacts of potential differences in steady‐state HVR responses (i.e., chemoreflex sensitivity), and subsequent differential renal responses on blood oxygenation and acid–base homeostasis between males and females at high altitude. Building on previous work, this study offers a unique high‐resolution data set that characterizes the respiratory, hematological, and renal responses to high altitude between males and females during rapid ascent and 10‐day residence to 3500 m. We hypothesized that (a) males will exhibit a larger steady‐state HVR and thus, a greater degree of acute respiratory alkalosis compared to females, (b) due to previously reported differences in renal tubular handling of bicarbonate in mammals, males will exhibit reduced renal compensation in response to respiratory alkalosis, and (c) these sex differences in renal‐respiratory acclimatization will manifest as differences in blood oxygenation and acid–base balance, with males having a more pronounced acid–base imbalance.

## 2 Methods and Materials

### 2.1 Ethics Statement

This study received ethical approval in advance from the University of Calgary Conjoint Human Research Ethics Board (Protocol REB24‐0083) and harmonized with the Mount Royal University Human Research Ethics Board (Protocol 103978). The recruitment and protocol were consistent with the Canadian Government Tri‐Council Policy on human research participants (TCPS2) and adhered to the standards of the Declaration of Helsinki, except for registration in a database.

### 2.2 Participant Recruitment

Healthy human female and male volunteers were recruited from members of a research expedition to La Paz, Bolivia. Consent of participants was voluntary, and members could choose to participate or not in any study without penalty, withdraw from an experiment at any time without justification or explanation, and exit the expedition at any time. Recruitment criteria included 18–60 years of age, non‐smoking, and non‐obese (body max index [BMI] < 32 kg/m^2^). Before recruitment, voluntary informed, verbal and written consent was obtained, and following pre‐screening for the inclusion/exclusion factors, expedition members were invited to partake in experimental testing sessions. Roughly equal numbers of female and male participants were recruited, and a comparison between sexes was planned a priori. Participants ate and drank water *ad libitum* throughout the expedition.

### 2.3 Measurements

#### 2.3.1 Study Protocol and Ascent Profile

Participants were tested for baseline values in Calgary, Alberta, Canada (1100 m) over a few days (D0). Participants were then flown to El Alto, Bolivia (4150 m), and transported by bus to the High Altitude Pulmonary and Pathology Institute in La Paz (3500 m), where they remained at this altitude for 10 days. Arterial blood draws were performed on participants on either exposure Days 2 or 3 (E‐HA; D2/3) and on exposure Days 9 or 10 (L‐HA; D9/10).

To further assess and confirm differences observed between males and females at 1100 m, P_a_CO_2_, [HCO_3_^−^]_a_, and pH_a_ were compiled from four separate studies (*n* = 80; 34 F, 46 M), including the present study (*n* = 38; 20 M; 18 F) and additional published data from our laboratory including data from arterial blood draws from Zouboules et al. (2018; *n* = 23; 13 M; 11 F), Bird et al. (2021; *n* = 17; 12 M; 5 F), and Russell et al. (2024; *n* = 28; 18 M; 10 F).

#### 2.3.2 Arterial Blood Draws

Arterial blood draws were performed by a trained and experienced physician (J.L.K.). An Allen's test of redundant hand circulation and/or participant preference was used to determine which radial artery was used. Arterial blood samples were acquired using a pre‐heparinized, self‐filling arterial blood syringe (PICO50, Radiometer, Mississauga, Ontario, Canada). All samples were immediately processed using a portable blood gas electrolyte machine (i‐STAT; Abbott, Mississauga, Ontario, Canada) and single‐use cartridges (CHEM8+ and CG4+) for analysis of S_a_O_2_, Hct, [Hb], C_a_O_2_ (using [Hb], P_a_O_2_ and S_a_O_2_), P_a_CO_2_, P_a_O_2_, pH_a_, [H^+^]_a_, [HCO_3_^−^]_a_, base excess (BEefc), total CO_2_ (TCO_2_), glucose, lactate, urea, creatinine, and anion gap. All samples were subject to local atmospheric pressure and thermal correction (37°C). To maintain consistency and reduce variability, the same research team members were responsible for handling and processing all blood samples (J.L.K., R.I., T.A.D.).

### 2.4 Data Analysis

To measure and report the partial pressure of inspired oxygen (P_I_O_2_) at altitude at each time point, we measured the atmospheric pressure daily via mercury manometer and the water vapor pressure of the respiratory tract (Sharma et al. 2022) using the following formula:

$$P_{I}O_{2}\left(\text{mmHg}\right)=\left(P_{\mathrm{ATM}}-47\right)\times \frac{21}{100}$$

where 47 is water vapor pressure in mmHg.

To assess oxygenation, oxygen content (C_a_O_2_) using [Hb] from the arterial blood draws and S_a_O_2_, using the following formula (Rhodes et al. 2022):

$$C_{a}O_{2}\left(\frac{\mathrm{mL}}{\mathrm{dL}}\right)=\left(1.34\times \left[\mathrm{Hb}\right]\times \left(\frac{S_{a}O_{2}}{100}\right)\right)+\left(0.0031\times P_{a}O_{2}\right)$$

where 0.0031 is the solubility constant of oxygen in blood (mL/mmHg·O_2_/dL) and 1.34 is the hemoglobin oxygen carrying capacity (mL·O_2_/g·Hb).

To assess ventilatory acclimatization, alveolar ventilation ($\dot{V}$_A_) was calculated from P_a_CO_2_ from arterial blood draws using the standard equation:

$${\dot{V}}_{A}\;\left(\frac{l}{\min}\right)=\frac{200\times 0.863}{P_{a}CO_{2}}$$

To characterize ventilatory acclimatization over time at altitude, the steady‐state chemoreflex drive (SSCD) was calculated using stimulus index (SI) and minute ventilation (${\dot{V}}_{A}$) in the following equation (Pfoh et al. 2017):

$$\text{SSCD}\left(a.u.\right)=\frac{{\dot{V}}_{A}}{\mathrm{SI}}$$

where $\text{stimulus index}\;\left(\text{mmHg}/\%\right)=\frac{P_{a}CO_{2\;}}{S_{a}O_{2}}$.

To assess hydration status and renal function, osmolality was calculated (Martín‐Calderón et al. 2015):

$$\text{Osmolality}=1.86\left(\left[Na^{+}\right]+\left[K^{+}\right]\right)+1.15\left(\frac{\mathrm{Glu}}{18}\right)+\frac{\text{Urea}}{6}+14$$

To evaluate the acid–base status of participants, the strong ion difference (SID) was calculated using the following formula (Stewart 1983; Chawla and Drummond 2008):

$$\mathrm{SID}=(\left[Na^{+}\right]+\left[K^{+}\right]+\left[Ca^{2+}\right]-\left(\left[\;\text{Lactate}\right]+\left[Cl^{-}\right]\right)$$

To calculate acid–base changes in blood, pH was calculated using the Henderson–Hasselbalch equation (Krapf et al. 1991):

$$pH_{a}=\mathrm{pKa}+\log\frac{{\left[\mathrm{HC}O_{3}^{-}\right]}_{a}}{s\times P_{a}CO_{2}}$$

where pKa=6.1 at $37^{{}^{\circ}}C$; $\;s=0.03\left(\text{mmol}\times L^{-1}\right)\times \mathrm{mmH}g^{-1}$

To evaluate pH_a_ compensation, the percent change in pH_a_ compensation is calculated using the following formula (Siesjö 1972):

$$\%-pH_{a}\;\text{compensation}=\frac{\Delta \left(pH_{a}\;\mathrm{at}\;3500m-\text{maximum}\;pH_{a}\right)}{\Delta \left(\text{control}\;pH_{a}\;\mathrm{at}\;1100m-\text{maximum}\;pH_{a}\right)}$$

where maximum pH was calculated using the Henderson–Hasselbalch equation using [HCO_3_^−^]_a_ at baseline and P_a_CO_2_ at altitude.

To analyze renal compensation, an index of renal reactivity (RR) was calculated using the following formula (Zouboules et al. 2018):

$$\mathrm{RR}=\frac{\Delta {\left[\mathrm{HC}O_{3}^{-}\right]}_{a}}{\Delta P_{a}CO_{2}}=\frac{{\left[\mathrm{HC}O_{3}^{-}\right]}_{a\;\text{altitude}}-{\left[\mathrm{HC}O_{3}^{-}\right]}_{a\;\text{baseline}}}{P_{a}C{O_{2}}_{\text{altitude}}-P_{a}C{O_{2}}_{\text{baseline}}}$$

where delta refers to the change in variable between 1100 and 3500 m.

To approximate the glomerular filtration rate (eGFR), creatinine levels from arterial blood draws were used in the following creatinine clearance (*C*_Cr_) formula (Inker et al. 2011):

$$\begin{array}{cc} C_{\mathrm{Cr}}\;\left(\frac{\mathrm{mL}}{\min}\right)\;=\; & 142\times {\left(\frac{S_{\mathrm{Cr}}}{A}\right)}^{B}\times 0.9938^{\mathrm{Age}}\\ & \times 1.012\;\left(\text{if female};\text{exclude for male}\right) \end{array}$$

where age is in years; $\text{serum creatinine}=S_{\mathrm{Cr}}\left(\frac{\mathrm{mg}}{\mathrm{dL}}\right)$ and *A* and *B* are constants used according to serum creatinine levels and sex as per Inker et al. (2011):

Female: $S_{\mathrm{Cr}}\leq 0.7,A=0.7,B=-0.241;\;S_{\mathrm{Cr}}>0.7,A=0.7,B=-1.2$.

Male: $S_{\mathrm{Cr}}\leq 0.9,A=0.9,B=-0.303;\;S_{\mathrm{Cr}}>0.9,A=0.9,B=-1.2$.

### 2.5 Statistical Approaches

To confirm statistical assumptions, we assessed normality of residuals using the Shapiro–Wilk test for each model; all variables met normality criteria, justifying the use of parametric statistical methods. In addition, one‐ (time) and two‐factor (time × sex) repeated‐measures ANOVAs were applied where complete data sets were available. In cases where there were missing data points, linear mixed models were applied. In all cases, statistically significant differences will be assumed at *p* < 0.05 (Graphpad Prism v10; CA, USA).

### 2.6 Statistical Analysis

For arterial blood draws, group participant data was reported at each time point: baseline (D0; 1100 m), E‐HA (D2/3) and L‐HA (D9/10) at high altitude (3500 m; i.e., three separate time points), and between females and males across all three time points.

#### 2.6.1 Group Responses

A mixed effects one‐factor repeated measures ANOVA analysis was performed to compare all values with the ascent for the whole group, comparing 1100 with 3500 m and any changes in daily values throughout the residence period. Where significant *F*‐ratios were detected, a Tukey's *post hoc* test was utilized for pairwise comparisons. Subsequently, to assess the change (delta) in variable responses, paired two‐tail *t*‐tests were performed.

#### 2.6.2 Sex Differences

To ascent between males and females, a mixed‐effect two‐factor ANOVA with repeated measures in values and non‐repeated measures in sex was used to compare 1100 and 3500 m, any changes in daily values throughout the residence period, as well as assessing the change (delta) in responses. Where significant *F*‐ratios were detected, a Tukey's *post hoc* test was utilized for pairwise comparisons.

#### 2.6.3 Statistical Corrections

For all repeated‐measures ANOVAs, the Geisser–Greenhouse correction was applied. Unpaired *t*‐tests using Welch's correction were performed when comparing sex differences, as well as the expanded participant pool to compare male and female P_a_CO_2_, [HCO_3_^−^]_a_, and pH_a_ at 1100 m.

#### 2.6.4 Statistical Limitations

The key outcome variables in this study are from single sample arterial blood draws across three time points, and across time in females and males. These measured and/or calculated metrics are often interrelated, representing the interaction between organ systems and their stimulus–response effects on arterial blood variables. However, the nature of these interactions raises the risk of statistical errors when multiple comparisons are carried out.

Tukey's HSD corrects for multiple pairwise comparisons within an individual model, but does not provide correction across the physiological outcomes analyzed in separate models. No additional multiplicity correction was applied across outcome variables. The outcomes included in the present study were selected a priori to characterize the interconnected physiological components underlying the broader ventilatory and acid–base responses to high‐altitude exposure described in our hypothesis, rather than as a collection of exploratory outcomes screened for statistical significance.

Several of these measures are also physiologically or mathematically interrelated and were therefore interpreted collectively to characterize the overall response to high altitude ascent and residence. Pairwise comparisons following a non‐significant interaction were performed for descriptive and exploratory purposes, and findings from these comparisons should be interpreted with appropriate caution regarding the increased risk of Type I error. Specifically, no correction was applied across the family of physiological outcomes analyzed in separate models. Therefore, given the number and interrelated nature of the outcomes examined, individual findings should be interpreted with consideration of the potential for increased Type I error. Pairwise comparisons were also performed following non‐significant interactions for descriptive and exploratory purposes. These comparisons were not interpreted as confirmatory evidence of an interaction, and should also be interpreted cautiously given the potential for increased Type 1 error (i.e., detecting a statistical difference when there is no effect or difference in the population).

## 3 Results

### 3.1 Participants

This data set included 38 participants where the group mixed‐effects analysis included repeated measures for all metrics (18 females, 20 males; Age = 34.5 ± 11.1 years; BMI = 26.1 ± 4.6 kg/m^2^). Sex difference mixed‐effects ANOVAs included repeated measures for time‐point and non‐repeated measures in sex (18 females, age = 32.1 ± 9.9 years, BMI = 25.6 ± 6.0 kg/m^2^; 20 males, age = 36.9 ± 11.9 years, BMI = 26.6 ± 2.1 kg/m^2^). No significant differences were detected between female and male age and BMI (*p* > 0.1667).

### 3.2 Group Analysis

The comparison of whole group ventilatory, blood gas, acid–base, and electrolyte values from baseline (D0), E‐HA (D2/3), and L‐HA (D9/10) are presented in Table 1.

**TABLE 1:** Comparison of ventilatory, blood gas, acid–base, and electrolyte data in all participants before (D0, 1100 m), at early high altitude following rapid ascent to 3500 m (D2/3), and late high altitude following 10‐day residence at 3500 m (D9/10). Atmospheric pressure, *P*_ATM_, was measured each day using a mercury barometer; partial pressure of inspired oxygen, P_I_O_2_, (calculated from *P*_ATM_); alveolar ventilation, $\dot{V}$_A_ (calculated from P_a_CO_2_); steady‐state chemoreflex drive, SSCD (see Section 2); partial pressure of arterial oxygen P_a_O_2_; arterial oxygen saturation, S_a_O_2_; arterial hemoglobin concentration, [Hb], arterial oxygen content, C_a_O_2_ (see Section 2); partial pressure of arterial carbon dioxide, P_a_CO_2_; arterial pH, pH_a_; arterial hydrogen ion concentration, [H^+^]_a_; arterial bicarbonate ion concentration, [HCO_3_^−^]_a_; base excess, BEecf; anion gap, AnGap; arterial strong difference index, SID (see Section 2); renal reactivity, RR (see Section 2); percent pH compensation, %pH_a_‐compensation (see Section 2); arterial osmolality (see Section 2), arterial creatinine; arterial lactate; glomerular filtration rate approximation, eGFR (see Section 2). *p* < 0.05 indicates a significant difference between baseline (*) or from previous time point (†), calculated using a mixed‐effects one factor ANOVA (*n* = 38).

Variable	D0	D2/3	D9/10	*p*
*P*_ATM_ (mmHg)	665.0	489.5 ± 2.1	489.5 ± 0.7	—
*P*_I_O_2_ (mmHg)	130.0	93.0	93.0	—
$\dot{V}$_A_ (L/min)	5.0 ± 0.5	5.7 ± 0.6*	5.94 ± 0.57*†	< 0.0001
SSCD	14.1 ± 3.1	17.4 ± 3.7*	18.5 ± 4.0*†	< 0.0001
P_a_O_2_ (mmHg)	84.2 ± 9.2	55.1 ± 5.2*	55.0 ± 5.1*	< 0.0001
S_a_O_2_ (%)	96.5 ± 1.4	89.1 ± 2.8*	89.6 ± 2.5*	< 0.0001
[Hb] (g/L)	141.4 ± 12.3	141.4 ± 10.7	148.4 ± 12.2*†	< 0.0001
C_a_O_2_ (mL/dL)	182.9 ± 15.3	170.0 ± 12.3*	178.3 ± 15.3*†	< 0.0001
P_a_CO_2_ (mmHg)	34.9 ± 3.6	30.3 ± 2.9*	29.3 ± 2.9*†	< 0.0001
pH_a_	7.43 ± 0.02	7.45 ± 0.02*	7.45 ± 0.02*	< 0.0001
[H^+^]_a_ (nM)	37.0 ± 1.8	35.0 ± 1.6*	36.0 ± 1.5*	< 0.0001
[HCO_3_^−^]_a_ (mmol/L)	23.3 ± 2.1	21.2 ± 2.2*	20.2 ± 1.8*†	< 0.0001
BEecf (mmol/L)	−0.9 ± 2.2	−2.7 ± 2.4*	−3.7 ± 1.9*†	< 0.0001
AnGap (mmol/L)	14.6 ± 1.5	16.2 ± 1.5*	14.3 ± 2.2†	< 0.0001
SID	37.4 ± 2.0	36.1 ± 2.2*	33.8 ± 2.1*†	< 0.0001
RR	—	0.29 ± 0.75	0.21 ± 1.83	0.7144
%‐pH_a_ compensation	—	50.0 ± 125.9	78.9 ± 40.8	0.0971
Osmolality (mmol/L)	292.7 ± 11.6	296.8 ± 18.3	290.8 ± 4.9	0.0643
Creatinine (umol/L)	71.4 ± 15.6	78.8 ± 15.5*	83.8 ± 22.3*	0.0002
Lactate (mmol/L)	0.9 ± 0.5	1.1 ± 0.5	0.9 ± 0.5	0.0535
eGFR (mL/min)	109.9 ± 15.6	102.5 ± 16.6*	98.1 ± 22.2*	0.0006

Figure 1 illustrates metrics of oxygenation for the whole group with rapid ascent (D2/3) and residence (D9/10) at 3500 m. P_a_O_2_ and S_a_O_2_ were significantly lower on D2/3 and D9/10 compared to D0 (*p* < 0.0001, Figure 1A,B). [Hb] was significantly higher D9/10 compared to D0 and D2/3 (*p* = 0.0003, Figure 1C). C_a_O_2_ significantly increased from D2/3 to D9/10 (*p* = 0.013, Figure 1D), but was not found to be different between D0 and D2/3. ΔP_a_O_2_ and ΔS_a_O_2_ calculated from D0 values were not different between ΔD2/3 and ΔD9/10 (*p* = 0.8749, *p* = 0.9014, Figure 1E,F). Δ[Hb] was significantly higher at ΔD9/10 compared to ΔD2/3 (*p* = 0.0010, Figure 1G). ΔC_a_O_2_ was not significantly different between ΔD2/3 and ΔD9/10 (*p* = 0.6363, Figure 1H).

**FIGURE 1:**
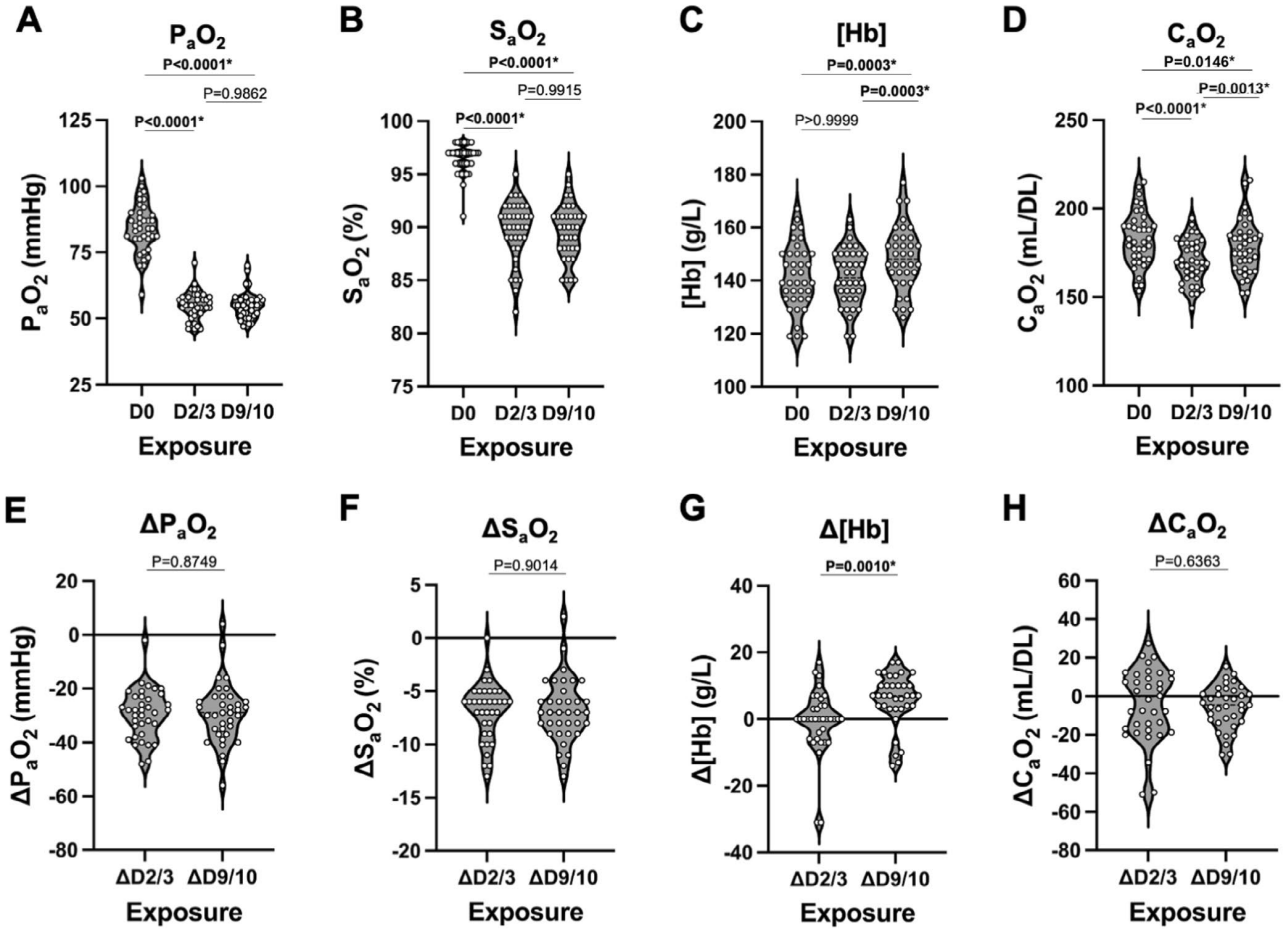
Comparison of blood gas and ventilation blood variables in participants (*n* = 38) at 3500 m. Individual data in participants illustrates the effect of high‐altitude exposure at 3500 m over 10 days. (A) Arterial partial pressure of arterial oxygen (P_a_O_2_). (B) Arterial oxygen saturation (S_a_O_2_). (C) Arterial hemoglobin concentration ([Hb]). (D) Calculated arterial oxygen content (C_a_O_2_). (E) Change (delta) in arterial partial pressure of oxygen relative to baseline (ΔP_a_O_2_). (F) Change in arterial oxygen saturation (ΔS_a_O_2_). (G) Change in arterial hemoglobin concentration (Δ[Hb]). (H) Change in arterial oxygen content (ΔC_a_O_2_). Mixed‐effects one factor ANOVA *p*‐values reported on each graph (A–D). Two‐tailed paired *t*‐test *p*‐values reported on each graph (E–H). Bold denotes significant differences (*p* < 0.05). Participants *n* = 38.

Figure 2 illustrates whole‐group metrics of acid–base homeostasis with rapid ascent (D2/3) and residence (D9/10) at high altitude. P_a_CO_2_ was significantly lower on D2/3 and D9/10 compared to D0 (*p* < 0.0001, Figure 2A) as well as significantly lower on D9/10 compared to D2/3 (*p* = 0.0062, Figure 2A). [HCO_3_^−^]_a_ was significantly lower on D2/3 and D9/10 compared to D0 (*p* = 0.0040, *p* < 0.0001, Figure 2B) as well as lower on D9/10 compared to D2/3 (*p* = 0.0202, Figure 2B). pH_a_ was significantly higher on D2/3 and D9/10 compared to D0 (*p* = 0.0020, *p* < 0.0001, Figure 2C). ΔP_a_CO_2_ and Δ[HCO_3_^−^]_a_ both significantly decreased at ΔD9/10 compared to ΔD2/3 (*p* = 0.0022, *p* = 0.0021, Figure 2D,E). ΔpH_a_ was not different between ΔD2/3 and ΔD9/10 (*p* = 0.2184, Figure 2F).

**FIGURE 2:**
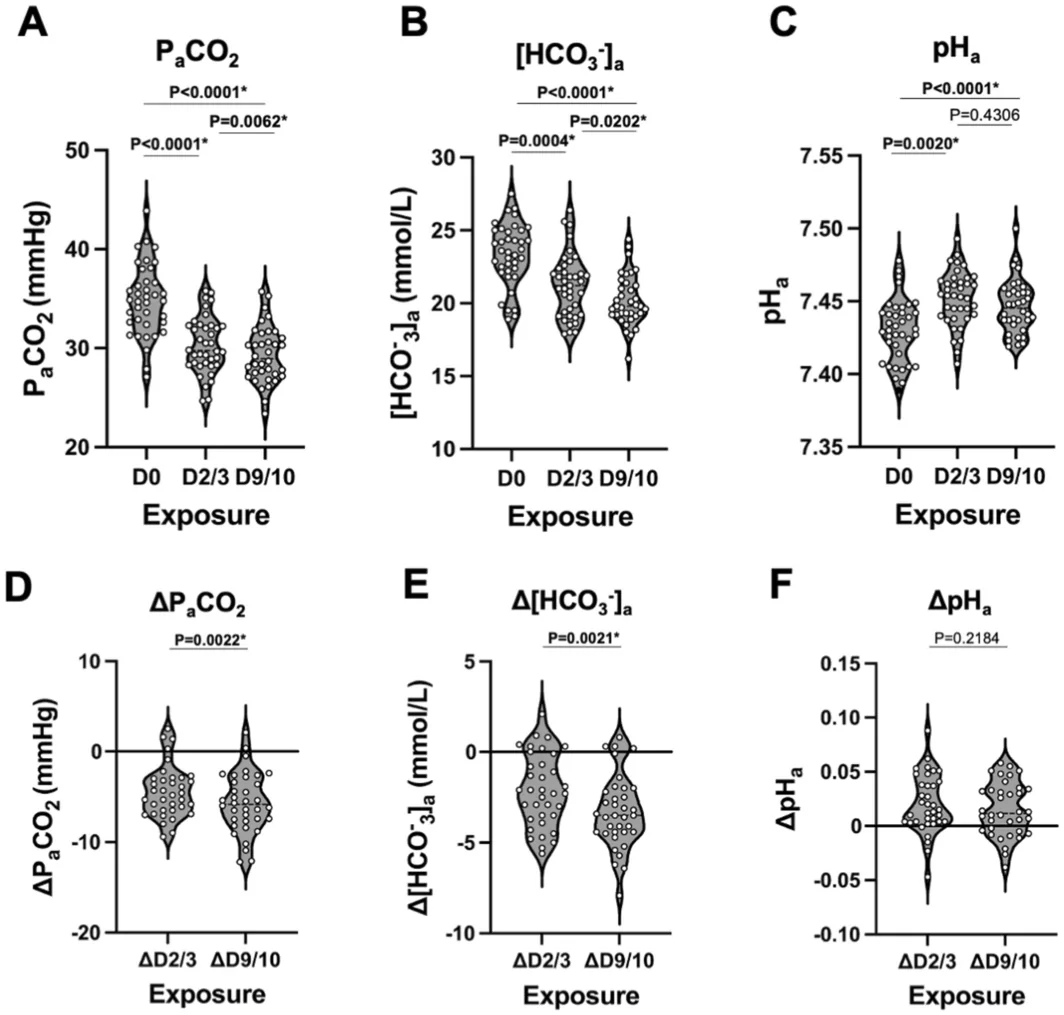
Comparison of blood gas, ventilation, and acid–base variables in participants (*n* = 38) at 3500 m. Individual data in participants illustrates the effect of high‐altitude exposure at 3500 m over 10 days. (A) Arterial partial pressure of arterial carbon dioxide (P_a_CO_2_). (B) Arterial bicarbonate ion concentration ([HCO_3_^−^]_a_). (C) Arterial pH (pH_a_). (D) Change (delta) in arterial partial pressure of carbon dioxide relative to baseline (ΔP_a_CO_2_). (E) Change in arterial bicarbonate ion concentration (Δ[HCO_3_^−^]_a_). (F) Change in arterial pH (ΔpH_a_). Mixed‐effects one factor ANOVA *p*‐values reported on each graph (A–C). Two‐tailed paired *t*‐test *p*‐values reported on each graph (D–F). Bold denotes significant differences (*p* < 0.05). Participants *n* = 38.

### 3.3 Sex Difference Analysis

Table 2 reports sex difference comparisons of ventilatory, blood gas, acid–base, and electrolyte values from D0, D2/3, and D9/10 including main effects (sex and altitude), as well as interaction effects. Of note, V̇_A_ was significantly higher in females at D0 and D2/3 (*p* = 0.0209, *p* < 0.0001), but the difference did not persist to D9/10 (*p* = 0.5267). SSCD was higher in females D0 and D2/3 (*p* = 0.0163, *p* = 0.0002), but this difference did not persist to D9/10 (*p* = 0.4367). [H^+^]_a_ was higher in females at D2/3 compared to males (*p* = 0.0469). BEecf was lower in females at D0 and D2/3 (*p* = 0.0136, *p* < 0.0001). SID was lower in females at D2/3 (*p* = 0.0011).

**TABLE 2:** Comparison of ventilatory, blood gas, acid–base, and electrolyte data in females and males before (D0, 1100 m), at early high altitude following rapid ascent to 3500 m (D2/3), and late high altitude following 10‐day residence at 3500 m (D9/10). Alveolar ventilation, $\dot{V}$_A_ (calculated from P_a_CO_2_); steady‐state chemoreflex drive, SSCD (see Section 2); partial pressure of arterial oxygen P_a_O_2_; arterial oxygen saturation, S_a_O_2_; arterial hemoglobin concentration, [Hb]; arterial oxygen content, C_a_O_2_ (see Section 2); partial pressure of arterial carbon dioxide, P_a_CO_2_; arterial pH, pH_a_; arterial hydrogen ion concentration, [H^+^]_a_; arterial bicarbonate ion concentration, [HCO_3_^−^]_a_; arterial base excess, BEecf; arterial anion gap, AnGap; arterial strong difference index, SID (see Section 2); renal reactivity, RR (see Section 2); percent pH compensation, %‐pH_a_ compensation (see Section 2); arterial osmolality (see Section 2); arterial creatinine; arterial lactate; glomerular filtration rate approximation, eGFR (see Section 2). *p* < 0.05 indicates a significant difference between baseline (*), from previous time point (†), or from males (~), calculated using a mixed effects two‐factor ANOVA (Female *n* = 18, Male *n* = 20).

		Exposure			*p*		
Variables	Sex	D0	D2/3	D9/10	Sex	Exposure	Interaction
$\dot{V}$_A_ (L/min)	Female	5.2 ± 0.6~	6.1 ± 0.5*~	6.0 ± 0.6*	0.0052	< 0.0001	0.0196
	Male	4.8 ± 0.4	5.4 ± 0.4*	5.8 ± 0.5*†			
SSCD	Female	15.4 ± 3.4~	19.6 ± 3.5*~	19.1 ± 4.4*	0.0044	< 0.0001	0.0383
	Male	13.0 ± 2.3	15.4 ± 2.6*	18.1 ± 3.6*†			
P_a_O_2_ (mmHg)	Female	86.6 ± 6.1	57.3 ± 5.5*	56.0 ± 5.1*	0.0165	< 0.0001	0.6121
	Male	82.1 ± 11.1	53.2 ± 4.1*	54.2 ± 5.2*			
S_a_O_2_ (%)	Female	96.9 ± 0.9	90.6 ± 3.0*	90.2 ± 2.7*	0.0313	< 0.0001	0.6925
	Male	96.1 ± 1.7	89.0 ± 2.5*	89.2 ± 2.4*			
[Hb] (g/L)	Female	133.3 ± 9.8~	135.6 ± 9.1~	141.1 ± 10.3*†~	< 0.0001	< 0.0001	0.3639
	Male	148.6 ± 9.7	146.6 ± 9.3	154.2 ± 10.4†			
C_a_O_2_ (mL/dL)	Female	173.4 ± 12.4~	164.6 ± 10.9*~	170.6 ± 12.3†~	0.002	< 0.0001	0.1569
	Male	191.5 ± 12.5	174.8 ± 11.6*	184.4 ± 14.8†			
P_a_CO_2_ (mmHg)	Female	33.5 ± 3.8~	28.5 ± 2.4*~	29.0 ± 3.1*	0.0077	< 0.0001	0.0204
	Male	36.1 ± 3.0	31.9 ± 2.3*	29.6 ± 2.7*†			
pH_a_	Female	7.43 ± 0.02	7.45 ± 0.02*~	7.45 ± 0.02*	0.1249	< 0.0001	0.2487
	Male	7.43 ± 0.02	7.46 ± 0.02*	7.45 ± 0.02			
[H^+^]_a_ (nM)	Female	38.0 ± 1.9	36.0 ± 1.7*~	36.0 ± 1.7*	0.1203	< 0.0001	0.2608
	Male	37.0 ± 1.7	35.0 ± 1.4 *	36.0 ± 1.4			
[HCO_3_^−^]_a_ (mmol/L)	Female	22.4 ± 2.4~	19.6 ± 1.3*~	20.0 ± 1.7*	0.0006	< 0.0001	0.0007
	Male	24.2 ± 1.5	22.6 ± 1.8*	20.4 ± 1.8*†			
BEecf (mmol/L)	Female	−1.8 ± 2.6~	−4.39 ± 1.4*~	−3.72 ± 2.0*	0.0011	< 0.0001	0.0003
	Male	0.0 ± 1.5	−1.2 ± 2.0*	−3.7 ± 2.0*†			
AnGap (mmol/L)	Female	15.1 ± 1.4	16.4 ± 1.8*	14.0 ± 2.5†	0.407	< 0.0001	0.2077
	Male	14.2 ± 1.5	15.9 ± 1.3*	14.5 ± 1.9†			
SID	Female	36.8 ± 2.1	34.9 ± 2.1*~	33.3 ± 2.6*	0.0034	< 0.0001	0.2216
	Male	37.9 ± 1.9	37.2 ± 1.8	34.3 ± 1.5*†			
RR	Female	—	0.24 ± 1.03	−0.26 ± 2.69	0.2431	0.5436	0.059
	Male	—	0.34 ± 0.38	0.60 ± 0.28			
%‐pH_a_ Compensation	Female	—	72.2 ± 39.6	73.1 ± 74.1	0.5593	0.1166	0.1186
	Male	—	30.1 ± 169.0	83.5 ± 45.0†			
Osmolality (mmol/L)	Female	292.9 ± 16.4	294.7 ± 15.9	289.8 ± 4.9	0.8071	0.0785	0.4211
	Male	292.6 ± 4.3	298.7 ± 20.5	291.7 ± 4.9			
Creatinine (mmol/L)	Female	60.7 ± 12.3~	69.3 ± 12.5*~	69.9 ± 19.1~	< 0.0001	0.0004	0.3481
	Male	81.1 ± 11.5	87.3 ± 12.8*	94.9 ± 18.4*			
Lactate (mmol/L)	Female	1.0 ± 0.5	1.3 ± 0.6~	1.0 ± 0.5	0.0197	0.0429	0.2575
	Male	0.8 ± 0.4	0.9 ± 0.4	0.8 ± 0.5			
eGFR (mL/min)	Female	113.1 ± 17.9	104.5 ± 19.8*	103.5 ± 25.0	0.2034	0.0009	0.5614
	Male	107.0 ± 12.9	100.6 ± 13.4*	93.8 ± 19.3*			

Figure 3 illustrates metrics of oxygenation with sex difference comparison during rapid ascent (D2/3) and residence (D9/10) at 3500 m. P_a_O_2_ was significantly higher in females on D2/3 (*p* = 0.0143, Figure 3A). [Hb] and C_a_O_2_ were higher in males at D0 (*p* < 0.001), D2/3 (*p* = 0.0008, *p* = 0.0085, Figure 3C,D), and D9/10 (*p* = 0.0007, *p* = 0.0045, Figure 3C,D). ΔP_a_O_2_ was not different between the sexes at ΔD2/3 and ΔD9/10 (*p* = 0.9094, *p* = 0.4548, Figure 3E) as well as no difference between females between ΔD2/3 and ΔD9/10 (*p* = 0.4126, Figure 3E) or males (*p* = 0.3749, Figure 3E). ΔS_a_O_2_ was not different between the sexes at ΔD2/3 and ΔD9/10 (*p* = 0.4132, *p* = 7440, Figure 3F) as well as no difference between females between ΔD2/3 and ΔD9/10 (*p* = 0.7052, Figure 3F) or males (*p* = 0.7426, Figure 3F). Δ[Hb] was not different between the sexes at ΔD2/3 and ΔD9/10 (*p* = 0.1435, *p* = 0.7390, Figure 3E), but was increased in males between ΔD2/3 and ΔD9/10 (*p* = 0.0034, Figure 3E). ΔC_a_O_2_ was larger in males ΔD2/3 (*p* = 0.0468, Figure 3H) as well as increased significantly in males between ΔD2/3 and ΔD9/10 (*p* = 0.0030, Figure 3H).

**FIGURE 3:**
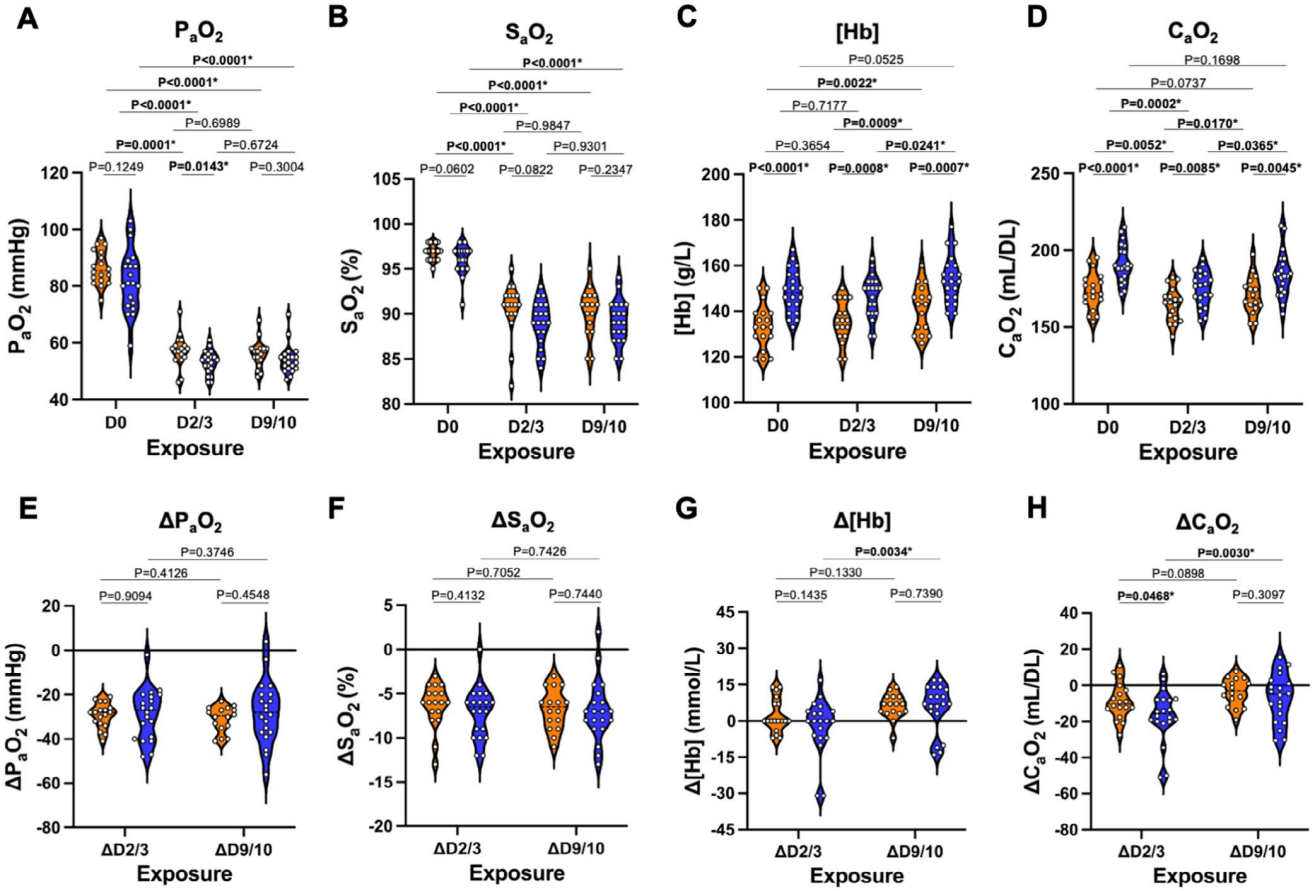
Comparison of blood gas and ventilation blood variables in females (orange) and males (blue) at 3500 m. Individual data in females and males illustrates the effect of high‐altitude exposure at 3500 m over 10 days. (A) Arterial partial pressure of arterial oxygen (P_a_O_2_). (B) Arterial oxygen saturation (S_a_O_2_). (C) Arterial hemoglobin concentration ([Hb]). (D) Calculated arterial oxygen content (C_a_O_2_). (E) Change (delta) in arterial partial pressure of oxygen relative to baseline (ΔP_a_O_2_). (F) Change in arterial oxygen saturation (ΔS_a_O_2_). (G) Change in arterial hemoglobin concentration (Δ[Hb]). (H) Change in arterial oxygen content (ΔC_a_O_2_). Mixed‐effects two‐factor ANOVA *p*‐values reported on each graph (A–H). Bold denotes significant differences (*p* < 0.05). Female *n* = 18, Male *n* = 20.

Figure 4 depicts metrics of acid–base homeostasis with sex difference comparison throughout rapid ascent (D2/3) and residence (D9/10) at high altitude. P_a_CO_2_ was significantly lower in females at D0 and D2/3 (*p* = 0.0280, *p* < 0.0001, Figure 4A). [HCO_3_^−^]_a_ was significantly lower in females D0 and D2/3 (*p* < 0.0001, *p* = 0.0119, Figure 4B). pH_a_ was lower in females on D2/3 (*p* = 0.0457, Figure 4C). ΔP_a_CO_2_ was not different between the sexes at ΔD2/3 and ΔD9/10 (*p* = 0.4322, *p* = 0.1129, Figure 4D), but was increased in males between ΔD2/3 and ΔD9/10 (*p* < 0.0001, Figure 4D). Δ[HCO_3_^−^]_a_ was not different between the sexes at ΔD2/3 and ΔD9/10 (*p* = 0.0533, *p* = 0.658, Figure 4E), but was increased in males between ΔD2/3 and ΔD9/10 (*p* < 0.0001, Figure 4E). ΔpH_a_ was not different between the sexes at ΔD2/3 and ΔD9/10 (*p* = 0.7031, *p* = 0.1193, Figure 4F), but was decreased in males between ΔD2/3 and ΔD9/10 (*p* = 0.0217, Figure 4F).

**FIGURE 4:**
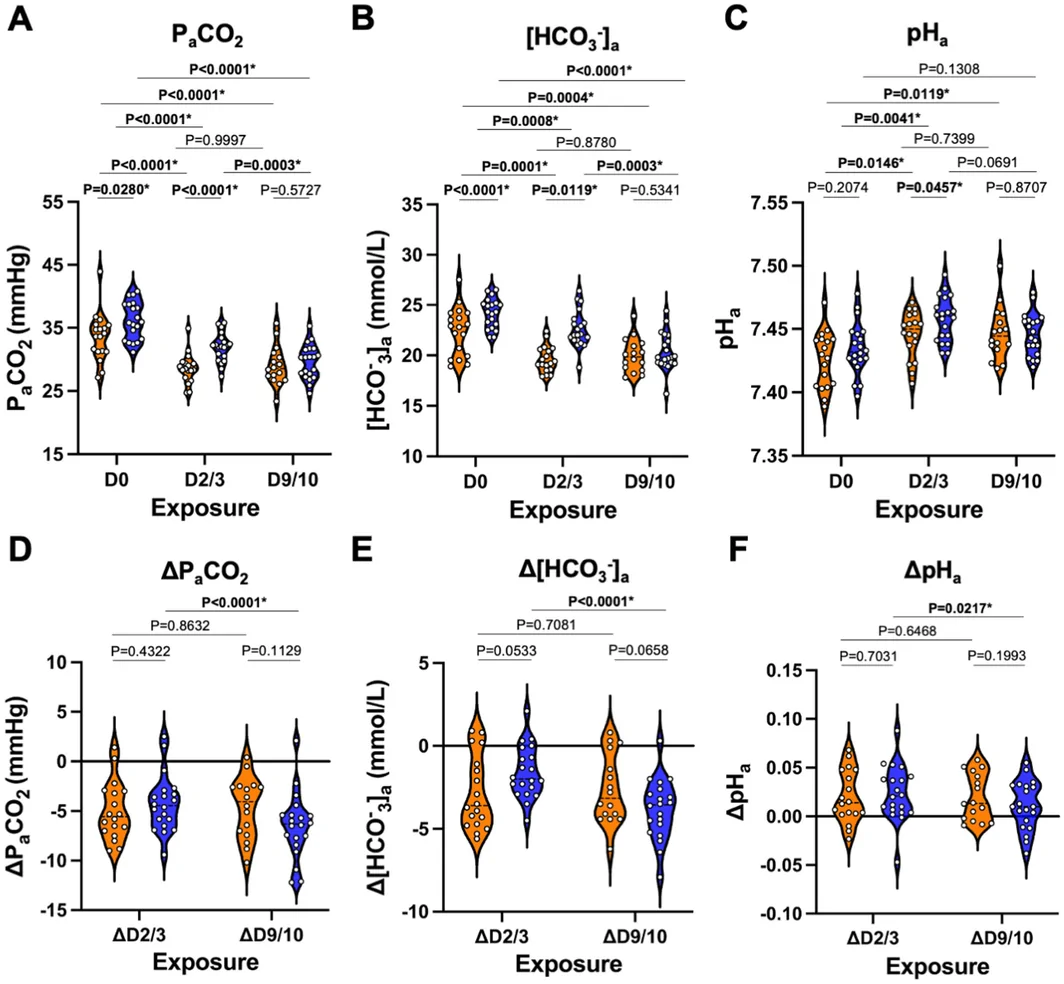
Comparison of blood gas, ventilation, and acid–base variables in females (orange) and males (blue) at 3500 m. Individual data in females and males illustrates the effect of high‐altitude exposure at 3500 m over 10 days. (A) Arterial partial pressure of arterial carbon dioxide (P_a_CO_2_). (B) Arterial bicarbonate ion concentration ([HCO_3_^−^]_a_). (C) Arterial pH (pH_a_). (D) Change (delta) in arterial partial pressure of carbon dioxide relative to baseline (ΔP_a_CO_2_). (E) Change in arterial bicarbonate ion concentration (Δ[HCO_3_^−^]_a_). (F) Change in arterial pH (ΔpH_a_). Mixed‐effects two‐factor ANOVA *p*‐values reported on each graph (A–F). Bold denotes significant differences (*p* < 0.05). Female *n* = 18, Male *n* = 20.

### 3.4 Davenport Diagrams

Figure 5 consists of Davenport diagrams that illustrate the qualitative integrated acid–base variables and metabolic compensation for females and males at baseline (D0, 1100 m). Figure 5A presents baseline values of females and males at 1100 m. Figure 5B shows the rightward and downward shift from baseline after rapid ascent to HA. Females showed a downward shift compared to males, indicating females had larger renal compensation upon rapid ascent (Figure 5B). Males had a more pronounced shift rightward compared to females, indicating a larger degree of alkalemia (Figure 5B). Figure 5C shows a slight leftward and downward shift in males from E‐HA to L‐HA, indicating later renal compensation in males (Figure 5B,C). However, females did not shift further, indicating that males mount a renal compensatory response over a larger time course compared to females (Figure 5B,C). Return to original baseline values was absent in both females and males, indicating only partial renal compensation was achieved for the whole group (Figure 5A *–*C).

**FIGURE 5:**
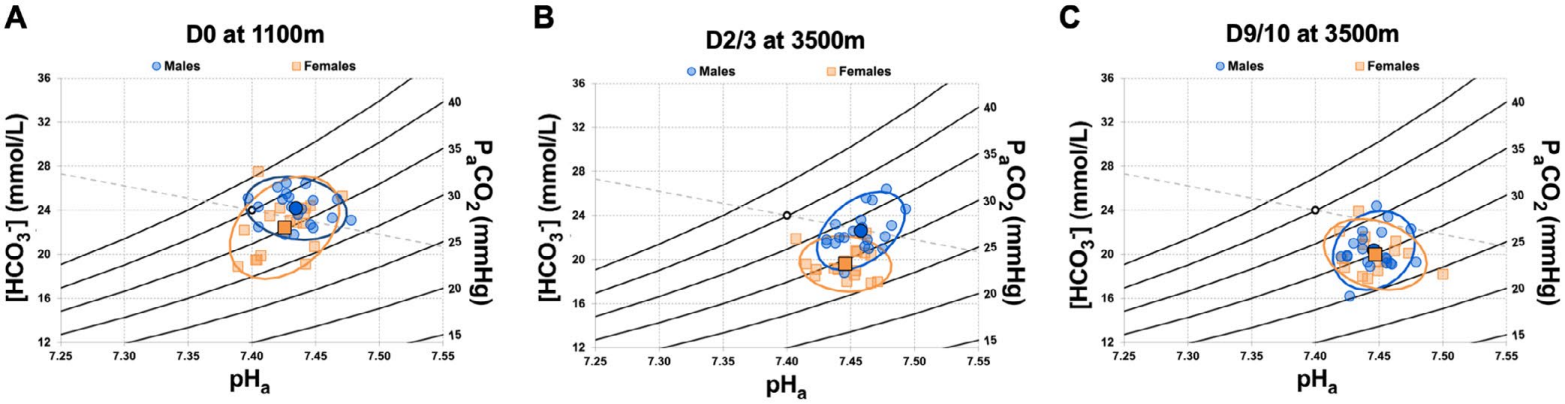
Davenport diagrams at 1100 and 3500 m after rapid ascent and over 10‐day residence at high altitude. Individual data of females (orange squares) and males (blue circles) demonstrating relationships between pH_a_, [HCO_3_^−^]_a_, and P_a_CO_2_, and at baseline (A), early high altitude (D2/3) (B), and late high altitude (D9/10) (C). Individual data points and group mean (larger symbols) depicted with 95% ellipse for each group. Arterial blood draws provided partial pressure of carbon dioxide (P_a_CO_2_), bicarbonate ion concentration ([HCO_3_^−^]_a_), and pH (pH_a_). Right hand *y*‐axis represents PaCO_2_ isopleths. Females *n* = 18, males *n* = 20.

### 3.5 Baseline Sex Difference Comparison

Figure 6 illustrates a comparison of blood gas and acid–base variables in a larger data set at 1100 m (*n* = 80; 34 F, 46 M). P_a_CO_2_ and [HCO_3_^−^]_a_ were significantly lower in females compared to males at 1100 m (*p* < 0.0001, Figure 6A,B). pH_a_ was not different between the sexes at 1100 m (*p* = 0.3213, Figure 6C), similar to previous reports (Crapo et al. 1999; Klæstrup et al. 2011).

**FIGURE 6:**
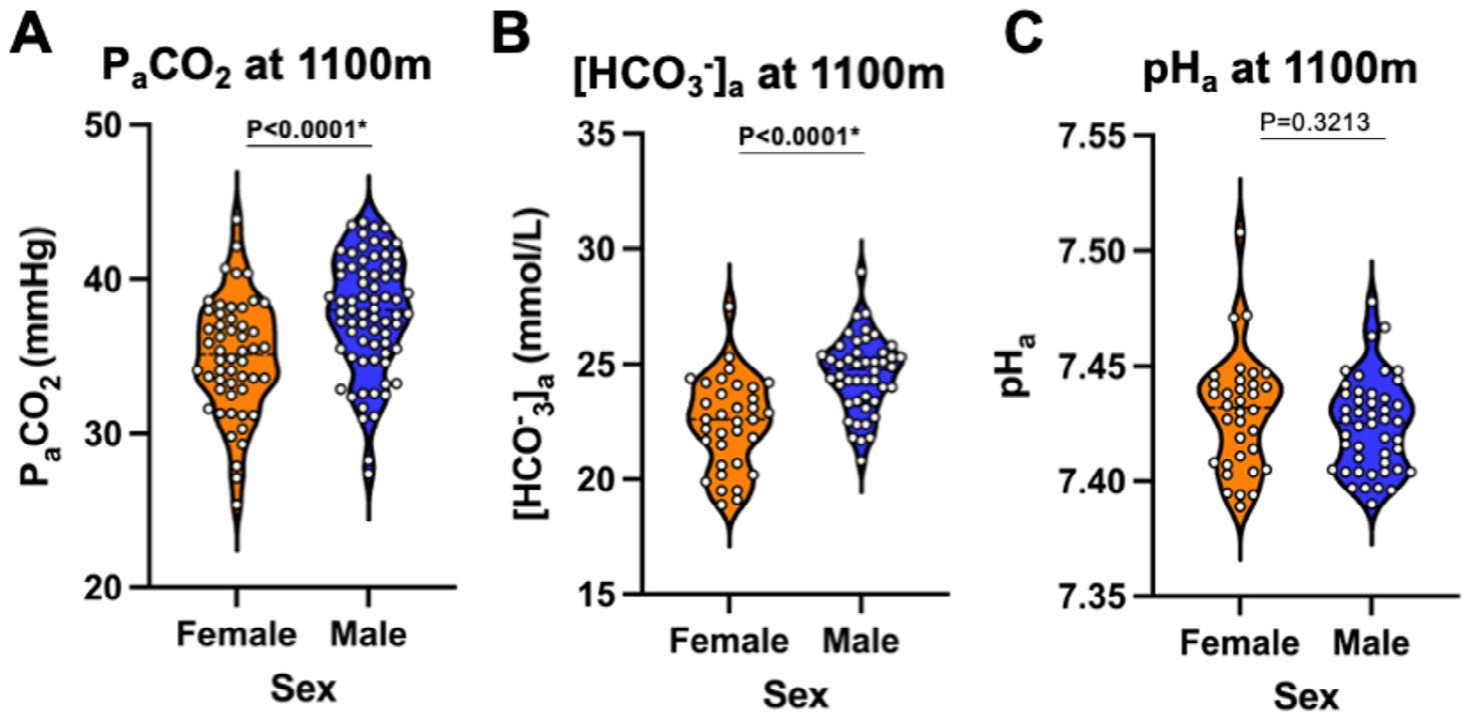
Comparison of blood gas and acid–base variables in a larger pooled sample of participants at 1100 m (*n* = 80; 34 F, 46 M) from the present and previous studies from our lab. Individual data in participants depicts sex‐based differential baseline acid–base variables. (A) Partial pressure of arterial carbon dioxide (P_a_CO_2_), whereby females were 33.56 ± 3.36 mmHg and males were 37.53 ± 3.29 mmHg (*p* < 0.0001). (B) Arterial bicarbonate ion concentration ([HCO_3_^−^]_a_), whereby females were 22.44 ± 1.98 mmol/L and males were 24.56 ± 1.63 mmol/L (*p* < 0.0001). (C) Arterial pH (pH_a_), whereby females were 7.43 ± 0.025 and males were 7.42 ± 0.20 (*p* = 0.3213). This sex difference in acid–base homeostasis at low altitude is in agreement with other published studies (e.g., Crapo et al. 1999; Klæstrup et al. 2011).

## 4 Discussion

We aimed to evaluate the impacts of potential differences in the HVR responses (i.e., chemoreflex sensitivity) and differential blood oxygenation and acid–base homeostasis between males and females with rapid ascent and a 10‐day residence at high altitude (3500 m). The principal findings were (a) females had significantly lower P_a_CO_2_ and [HCO_3_^−^]_a_ at low altitude baseline, 1100 m (*p* < 0.0001), (b) at early high altitude (E‐HA; D2/3) females had significantly higher $\dot{V}$_A_ and P_a_O_2_ (*p* < 0.0001, *p* = 0.0143) and significantly lower P_a_CO_2_, [HCO_3_^−^]_a_, and pH_a_ compared to males (*p* < 0.0001, *p* = 0.119, *p* = 0.0457), and (c) at late high altitude (L‐HA; D9/10) no difference existed between female and male $\dot{V}$_A_, P_a_O_2_, P_a_CO_2_, [HCO_3_^−^]_a_, and pH_a_ (*p* ≥ 0.3004). These findings suggest that females have a more rapid ventilatory and renal response than males. However, males eventually “catch up” to females, indicating a larger time course is required to mount the male compensatory renal response. To our knowledge, this study is the first to compare ventilatory and renal acid–base acclimatization in females and males following rapid ascent and residence to high altitude, with a clear demonstration of females exhibiting faster and more pronounced responses to high altitude. This response is likely driven in part by females baseline lower P_a_CO_2_ and [HCO_3_^−^]_a_, and more pronounced bicarbonate excretion, which may mediate enhanced compensation to the stressors of high altitude‐mediated acid–base disturbances.

### 4.1 Baseline Differences

Baseline differences between males and females in P_a_CO_2_ and [HCO_3_^−^]_a_ may result from differential patterns in renal anatomy and function between sexes. Sexually dimorphic patterns in the expression of multiple renal epithelial sodium transporters, which correlate with altered responses to salt homeostasis, have been observed in rodents (Veiras et al. 2017). For instance, computational models of rat proximal convoluted tubules indicate that fractional sodium reabsorption is lower in females due to a smaller transport area and reduced expression of sodium‐hydrogen ion exchanger (NHE3) and calbindin‐2 (Li et al. 2018). Veiras et al. (2017) identified that female mice, in response to a fast, had reduced bicarbonate ion reabsorption compared to male mice. This is congruent with previous literature identifying differential patterns in serum [HCO_3_^−^] levels between sexes in healthy human adults (Duan et al. 2023; Ibrahim et al. 2023). Pre‐menopausal females were found to have decreased [HCO_3_^−^]_a_ levels compared to males (Duan et al. 2023; Ibrahim et al. 2023). Duan et al. (2023) performed a cross‐sectional cohort study involving over 93,000 patients, revealing that [HCO_3_^−^]_a_ levels are lower in females than males until approximately 50 years of age. From 35 to 50, [HCO_3_^−^]_a_ levels in females gradually increase, eventually surpassing males post‐menopause (Duan et al. 2023). This change aligns with hormonal shifts, particularly the decline in progesterone and estrogen concentrations during menopause (Hodgkinson 1982). In addition, pre‐menopausal females on oral contraceptives exhibited lower [HCO_3_^−^]_a_ levels compared to those not using contraceptives, implicating an effect of sex hormones in bicarbonate regulation (McPherson et al. 1978).

Progesterone is a known respiratory stimulant (e.g., Dempsey et al. 1986; Behan and Kinkead 2011; Boukari et al. 2015). Importantly, baseline P_a_CO_2_ is known to be lower in females than males, a difference that has been consistently observed at both sea level and high altitude (e.g., Fitzgerald and Haldane 1905; M. P. Fitzgerald 1913; Loeppky et al. 2001). Thus, prior to menopause, higher progesterone levels in females may stimulate steady‐state hyperventilation relative to males (Hodgkinson 1984). Loeppky et al. (2001) demonstrated that despite these baseline differences, women and men show similar ventilatory responses to acute altitude exposure, reinforcing the idea that progesterone enhances baseline ventilation rather than altitude‐induced ventilatory plasticity. This subtle relative hyperventilation in resting ventilation likely contributes to lower [HCO_3_^−^]_a_ levels due to reduced blood P_a_CO_2_, given that the majority of CO_2_ is carried in blood as [HCO_3_^−^]. Estradiol (serum estrogen) potentiates this effect by increasing progesterone receptor expression (Leavitt and Blaha 1972; LoMauro and Aliverti 2015). The loss of these effects post‐menopause is likely due to declining estrogen and progesterone levels, thus likely contributing to the observation of a rise in [HCO_3_^−^]_a_ in females post‐menopause (Duan et al. 2023). The respiratory effects of progesterone are mediated through complex mechanisms involving at least eight receptors, including nuclear and membrane forms, in which the relative roles of respiratory control are only partially understood (Boukari et al. 2015; Boukari et al. 2016; Marcouiller et al. 2014).

Our finding of lower PaCO_2_ and [HCO_3_^−^]a in females is consistent with previous findings (e.g., Crapo et al. 1999; Klæstrup et al. 2011), as well as in an expanded data set compiled from four separate studies from our group (*n* = 80; 34 F, 46 M; see Figure 6), where arterial blood PCO_2_ and [HCO_3_^−^] were compared between sexes, with both variables significantly lower in females. This is consistent in the data set for this study, where the arterial P_a_CO_2_ and [HCO_3_^−^]_a_ were both significantly lower in females (*p* = 0.0280, *p* < 0.0001, Figure 4A,B). The mean age of the female participants in this study was 32.1 ± 9.9 years and thus, consisted primarily of pre‐menopausal females. This result aligns with prior findings that lower P_a_CO_2_ in females is a persistent trait rather than solely a function of altitude exposure (e.g., Fitzgerald and Haldane 1905). Thus, increased ventilation results in reduced P_a_CO_2_ and respiratory alkalosis, leading to reduced [HCO_3_^−^]_a_ due first to the bicarbonate buffer system, and secondarily to compensatory renal compensation (e.g., Johnson, Dickenson, et al. 2025; Johnson, Strzalkowski, et al. 2025). In females, [HCO_3_^−^]_a_ is further decreased due to differential renal handling (i.e., excretion).

### 4.2 Ventilatory Acclimatization

Ventilatory acclimatization can be assessed through numerous methods following ascent. Transient tests of the HVR are regularly used in laboratory contexts, whereby the transient respiratory response to an acute inspired hypoxic exposure is quantified (e.g., Steinback and Poulin 2007; Pfoh et al. 2017; Oeung et al. 2023). A review and meta‐analysis performed by Oeung et al. (2023) compared all known studies previously used to quantify the HVR in humans using acute tests (e.g., transient, step‐down, rebreathing). Their findings suggest that regardless of the test or metric used, the HVR values were highly variable and low in magnitude (i.e., positively skewed; Oeung et al. 2023), unless acclimatized, whereby response magnitudes were higher (i.e., ventilatory acclimatization). However, the results of transient hypoxic tests are cofounded for a myriad of reasons, and thus may not capture the steady‐state ventilatory response accurately (e.g., Pfoh et al. 2017). Accordingly, steady‐state resting ventilation and/or arterial or end‐tidal CO_2_ can be used to assess the steady‐state ventilatory response to high‐altitude exposure (e.g., Hackett et al. 1980; Sun et al. 1990; Zhuang et al. 1993; Curran et al. 1997; Beall 2007; Slessarev et al. 2010). Another more recently developed metric called SSCD quantifies the steady‐state ventilatory response to prevailing chemostimuli (Pfoh et al. 2017) and appears to capture ventilatory acclimatization with ascent to high altitude (Cates et al. 2022; Johnson, Dickenson, et al. 2025; Johnson, Strzalkowski, et al. 2025; Leacy et al. 2021). Bird et al. (2021) conducted a similar study to the present, where participants underwent arterial blood draws at baseline, and at early and late ascent to high altitude following rapid ascent and 9‐day residence at 3800 m. Bird et al. (2021) showed that both minute ventilation ($\dot{V}$_E_) and SSCD increased upon immediate ascent to 3800 m, and both further increased by 9 days of residence at 3800 m, and that ventilatory acclimatization was responsible for further hypocapnia throughout residence at high altitude (Bird et al. 2021). In the present study, alveolar ventilation ($\dot{V}$_A_; calculated from P_a_CO_2_; see Section 2) and SSCD also increased upon ascent to 3500 m, as well as increased throughout residence at high altitude (*p* < 0.001, Table 1). Therefore, as expected upon rapid ascent to 3500 m in the present study, the HVR response was elicited, and ventilatory acclimatization was responsible for the increased hypocapnia throughout the residence at 3500 m.

### 4.3 Renal Acclimatization and Acid–Base Regulation

In response to ventilatory‐induced hypocapnia and subsequent respiratory alkalosis, we anticipated a subsequent compensatory metabolic acidosis, characterized by a decrease in [HCO_3_^−^]_a_ in all participants (e.g., Zouboules et al. 2018; Bird et al. 2021; Johnson, Dickenson, et al. 2025). Participants showed a significant decrease in [HCO_3_^−^]_a_ following ascent to 3500 m (*p* = 0.0004). However, participants remained alkalemic at E‐HA (D2/3, *p* = 0.0020) as well as L‐HA (D9/10, *p* < 0.0001), indicating that only partial renal compensation was achieved for our group during the 10‐day residence at 3500 m.

Incomplete renal compensation after 10 days of HA exposure is both congruent and incongruent with previous literature. Forster et al. (1975) and Steele et al. (2022) both assessed longitudinal acid–base regulation upon rapid ascent and residence to 4300 m, where alterations in P_a_CO_2_ [HCO_3_^−^]_a_, and pH_a_ were investigated over 11 days (Forster et al. 1975) and 21 days (Steele et al. 2022). Similar to our study, both found pH_a_ compensation to be incomplete throughout the entire high altitude residence period, which was thought to be caused by disproportionate reductions in both P_a_CO_2_ and [HCO_3_^−^]_a_. Additionally, Johnson, Dickenson, et al. (2025), who investigated oxygenation and acid–base differences in acclimatization of indigenous Tibetan highlanders versus native lowlanders, found that upon incremental ascent to 4300 m, lowlanders remained alkalemic after 3–4 days of acclimatization at 4300 m. However, Bird et al. (2021) found pH_a_ returned to normal values within 24 h following ascent to 3800 m, and Zouboules et al. (2018) found that pH was normalized back to baseline values following one‐night residence at 4300 m. These disparate findings highlight the inherent variability in acid–base homeostatic regulation across a variety of participant groups and ascent profiles.

The present study illustrates incomplete renal compensation, likely due to the relative severity of the hypocapnic response. Bird et al. (2021) suggest that there may be a threshold for the efficacy of renal compensation mitigating P_a_CO_2_ disturbances, with previous work indicating this threshold exists between 3800 and 4300 m. However, the inability of participants in this study to achieve full renal compensation at 3500 m suggests that this threshold may be as low as 3500 m. These findings highlight the variability in acid–base homeostasis and limitations of renal compensation at altitude, particularly in response to more severe hypocapnic stimuli experienced with higher altitudes.

### 4.4 Sex Differences in Ventilatory Acclimatization

Our study comprised a participant pool with almost equal distribution of males and females (*n* = 38; 20 M; 18 F), allowing the novel comparison of sex differences in ventilation and acid–base acclimatization before and during rapid ascent and residence at high altitude. At baseline, females had significantly lower P_a_CO_2_ compared to males, likely due to circulating progesterone acting as a steady‐state respiratory stimulant. Since pre‐menopausal females have elevated progesterone levels compared to males, it is reasonable to conclude that females have higher resting ventilation and concomitant lower P_a_CO_2_ (*p* = 0.0280, Table 2 and Figure 4A). This result is also consistent with calculated alveolar ventilation (*V̇*_A_) being significantly higher in females at baseline and E‐HA (*p* = 0.0209, *p* < 0.0001). Increased ventilation also resulted in P_a_O_2_ being significantly higher in females at E‐HA (D2/3) (*p* = 0.0143, Table 2 and Figure 3A). Upon ascent to 3500 m, P_a_CO_2_ remained significantly lower in females than in males (*p* < 0.0001, Table 2 and Figure 4A). However, the ΔP_a_CO_2_ was not significantly different between males and females at E‐HA (*p* = 0.4322, Figure 4D). This indicates that males and females did not significantly differ in their relative P_a_CO_2_ reductions with ascent, but rather females maintained the relatively higher minute ventilation and lower P_a_CO_2_ with ascent.

These findings align with previous work reporting that females exhibit persistently lower P_a_CO_2_ levels compared to males at low altitude (e.g., Crapo et al. 1999; Klæstrup et al. 2011) and during initial high‐altitude exposure (e.g., M. P. Fitzgerald 1913, 1914; Loeppky et al. 2001). Fitzgerald's early studies at various altitudes documented sustained sex differences in respiratory drive, while Loeppky et al. (2001) further demonstrated that even at HA, females maintain lower P_a_CO_2_ despite similar ventilatory acclimatization responses as males. However, our study suggests that this difference does not persist with prolonged acclimatization, as by L‐HA, there was no significant difference in P_a_CO_2_ between sexes (*p* = 0.5727, Figure 4A). This is consistent with the findings of Cudkowicz et al. (1972) who demonstrated no difference in P_a_CO_2_ in lowlander females vs. males following a 3‐week residence at 3600 m. These findings highlights the dynamic nature of ventilatory acclimatization, and suggests that sex‐based differences in baseline P_a_CO_2_ may diminish with time at altitude, when ventilatory acclimatization persists.

[Hb] was significantly higher in males at all time points (Table 2 and Figure 3C). This was expected, as males are known to have higher hemoglobin mass than females (e.g., Vahlquist 1950; Yip et al. 1984). This sex difference is considered to be caused by the stimulatory effect of androgens on erythropoietin production in the kidney in males, and an inhibitory effect of estrogen on erythrocytosis in females (Jelkmann 2011; Shahani et al. 2009). Testosterone also has a synergistic effect with androgens on erythrocytosis, resulting in increased erythropoiesis (Murphy 2014). However, females do not attempt to compensate for their lower hemoglobin levels by increasing erythropoietic drive (Tilling et al. 2013). Thus, females maintain their [Hb] at a lower level than adult males at a different homeostatic steady‐state (Murphy 2014). Given C_a_O_2_ has contributions from P_a_O_2_, S_a_O_2_, and [Hb] (see Section 2), C_a_O_2_ is higher in males at every time point due to increased [Hb], despite lower P_a_O_2_ (Table 2 and Figure 3D).

### 4.5 Sex Differences in Time Course, Magnitude, and Variability of Renal Responses

[HCO_3_^−^]_a_ was significantly lower in females at baseline and E‐HA (D2/3) when compared to males (*p* < 0.0001, *p* = 0.0119, Table 2 and Figure 5B). However, Δ[HCO_3_^−^]_a_ was not different between females and males at E‐HA (*p* = 0.0533), indicating that the reduction in [HCO_3_^−^]_a_ was not significantly different between the sexes upon rapid ascent. Although the reduction was not significantly different, females having reduced [HCO_3_^−^]_a_ at baseline are at a better starting point for mounting the compensatory metabolic acidosis with ascent, and thus, are potentially better at buffering pH_a_ upon rapid ascent. This is evident when assessing absolute pH_a_ values. At baseline, pH_a_ is not different between the sexes (*p* = 0.2074, Table 2 and Figure 4C), but upon rapid ascent to HA, pH_a_ is significantly higher in males (*p* = 0.0457, Table 2 and Figure 4C). Thus, males were significantly more alkalemic than females with early ascent, indicating that females respond more rapidly with exposure to high altitude from both ventilatory and renal perspectives. This finding contrasts with the results of Loeppky et al. (2001), who observed that females exhibited higher pH_a_ than males after 12 h of high altitude exposure. However, this discrepancy is likely due to differences in study duration. While Loeppky et al. (2001) examined responses within a short‐term exposure period (hours), our findings demonstrate that over multiple days at 3500 m, males exhibit a greater alkalemic response, with higher pH_a_ values at E‐HA. We speculate this enhanced response is in part due to females already having reduced P_a_CO_2_ and [HCO_3_^−^]_a_ and thus having a “head start” compared to males in renal compensation to the respiratory alkalosis.

This conclusion is further confirmed when assessing the time course in the female vs. male renal response. Males are significantly more alkalemic compared to females at E‐HA. However, this difference does not persist to L‐HA. As there are no significant differences in P_a_CO_2_, [HCO_3_^−^]_a_, or pH_a_ between females and males by L‐HA (D9/10) (*p* = 0.5727, *p* = 0.5341, *p* = 0.8707, Table 2 and Figure 4). ΔP_a_CO_2_, Δ[HCO_3_^−^]_a_, and ΔpH_a_ were all significantly different in males between ΔD2/3 and ΔD9/10 (*p* < 0.0001, *p* < 0.0001, *p* = 0.0270, Figure 4). These different delta responses indicate that males are mounting their renal compensatory response on a longer time course (i.e., between exposure D2/3 and D9/10), which is further confirmed by male absolute P_a_CO_2_ and [HCO_3_^−^]_a_, being significantly lower at L‐HA compared to E‐HA (*p* = 0.0003, Figure 4A,B) as well as being no different from female P_a_CO_2_ and [HCO_3_^−^]_a_ by L‐HA (*p* = 5727, *p* = 0.5341, Figure 4A,B) This is different than what was observed in females as ΔP_a_CO_2_, Δ[HCO_3_^−^]_a_, and ΔpH_a_ was not significantly different between ΔD2/3 and ΔD9/10 (*p* ≥ 0.6468, Figure 4). The more rapid response is apparent in females as P_a_CO_2_, [HCO_3_^−^]_a_, are significantly lower upon rapid ascent and early exposure to HA (D2/3) (*p* < 0.0001, Figure 4A,B) as well as pH_a_ is significantly higher (*p* = 0.0146, Figure 4C). P_a_CO_2_, [HCO_3_^−^]_a_, or pH_a_ was not different between E‐HA and L‐HA (*p* ≥ 0.7399, Figure 4), indicating female response to HA did not change between early and late altitude exposure, thus indicating a rapid renal compensation upon rapid ascent and residence at high altitude. Therefore, we conclude that females had a more rapid and larger magnitude renal response than males upon ascent to and residence at 3500 m.

Davenport diagrams are graphical qualitative demonstrations of the Henderson‐Hasselbalch relationship before, during, and after an acid–base disturbance and compensation. They demonstrate the respiratory alkalosis (i.e., the acid–base disturbance) and the corresponding metabolic acidosis (compensation), depicted by P_a_CO_2_ isopleths, [HCO_3_^−^]_a_, pH_a_, and the non‐HCO_3_^−^ buffer slope (Davenport 1974). Figure 6D illustrates the Davenport diagram of females and males at 1100 m in the expanded data set (*n* = 123). Females have a slight downward shift compared to males, thereby visualizing decreased female P_a_CO_2_ and [HCO_3_^−^]_a_ levels at 1100 m (Figure 6D). Figure 5 consists of three Davenport diagrams which illustrate the metabolic compensation to the respiratory alkalosis between females and males at 1100 m (Figure 5A), then at E‐HA (D2/3) at 3500 m (Figure 5B), and L‐HA (D9/10) at 3500 m (Figure 5C). In both females and males, there was a significant decrease in P_a_CO_2_ (the respiratory disturbance) and [HCO_3_^−^]_a_ (renal compensation) and a significant increase in pH_a_ after ascent to 3500 m (Figure 5B). Males had a more significant rightward shift than females, thereby illustrating their more severe alkalemia. E‐HA females had a more severe downward shift than males, displaying their lower P_a_CO_2_ and [HCO_3_^−^]_a_ (Figure 5B). However, by L‐HA (D9/10), males experienced a slightly leftward and downward shift, similar to female P_a_CO_2_, [HCO_3_^−^]_a_, and pH_a_ values (Figure 5A). This shift further demonstrates that male renal compensation takes place on a longer time course compared to females.

### 4.6 Potential Underlying Mechanisms

As discussed previously, progesterone is a respiratory stimulant that is known to decrease resting P_a_CO_2_ in females compared to males, and thus, pre‐menopausal females would be expected to have proportionately reduced P_a_CO_2_ and [HCO_3_^−^]_a_ levels. In a previous study characterizing acute hypoxia‐mediated hyperventilation, a reduction in P_a_CO_2_ of −13.5% led to a chemically driven proportional reduction in [HCO_3_^−^]_a_ of approximately −3.7% during acute, short‐duration hypoxic exposure to 0.12 F_I_O_2_ (Johnson, Strzalkowski, et al. 2025). However, when the percent reduction was calculated between males and females in our larger data set at 1100 m, females have an approximately −7.4% lower P_a_CO_2_, but an approximately −7.1% lower [HCO_3_^−^]_a_ compared to males. This difference implicates another mechanism in addition to “chemistry” accounting for lower baseline [HCO_3_^−^]_a_ in females, likely differences in renal handling. In addition, to approximate the glomerular filtration rate (eGFR), creatinine levels from arterial blood draws were used (see Section 2). We found no differences in eGFR between sexes at 1100 m, E‐HA, or L‐HA exposure (Table 2). In conjunction with the demonstrated reduction of [HCO_3_^−^]_a_ in females, this result suggests the sex differences demonstrated here are isolated to renal electrolyte transport in renal tubules, rather than differences in glomerular filtration.

Evidence indicates sex differences in renal handling of HCO_3_^−^ is likely influenced by hormonal regulation. A previous study has identified sex differences in RNA expression in renal SLC transporters, a family of anion transporters crucial for bicarbonate handling (Huang et al. 2020). Females were associated with reduced RNA SLC expression (Huang et al. 2020). However, this difference did not translate to dimorphic protein expression (Huang et al. 2020). Despite this, females still exhibit renal tubule organization that favors reduced HCO_3_^−^ reabsorption (Brasen et al. 2014; Veiras et al. 2017). Specifically, females demonstrate greater phosphorylation (i.e., inactivation) of sodium‐hydrogen exchanger isoform 3 (NHE3), localized at the base of microvilli, along with lower expression of Na^+^/Pi cotransporters and aquaporins, which collectively reduces bicarbonate reabsorption capacity within the proximal convoluted tubules (Veiras et al. 2017). These differences are further reflected in urine pH, as female urine is typically more alkaline than males, indicating reduced HCO_3_^−^ retention and, thus, increased alkali absorption in males (Worcester et al. 2018). Worcester et al. (2018) hypothesized that reduced HCO_3_^−^ retention may be evolutionarily advantageous for females, potentially linked to maintaining bone mineral density during pregnancy and lactation when electrolyte demands are elevated. Furthermore, sex hormones play distinct roles in regulating transporter abundance along different nephron segments, with androgens primarily influencing the proximal tubular transport, and estrogens affecting distal tubule function (McDonough et al. 2023). Such hormonal and structural differences are said to prepare females for unique fluid and electrolyte demands faced throughout the life cycle (McDonough et al. 2023). Accordingly, these hormonal differences appear to also influence the rapid compensatory response to the respiratory alkalosis that accompanies high altitude exposure in females.

Male renal compensation occurring over a longer time course is evident through their significantly more alkalemic pH_a_ at E‐HA than females (*p* = 0.0475, Table 2 and Figure 4C). However, by L‐HA, male pH_a_ decreased to mirror that of females (*p* = 0.8707, Table 2 and Figure 4C). This longer time course is likely due to the increased ability for HCO_3_^−^ reabsorption in males compared to females (Brasen et al. 2014; Veiras et al. 2017; Worcester et al. 2018; McDonough et al. 2023). For males, with higher baseline [HCO_3_^−^]_a_ levels and a slower reduction in P_a_CO_2_ upon ascent, renal compensation required more time to reduce [HCO_3_^−^]_a_ and restore pH_a_ adequately. Slower reduction in P_a_CO_2_ is highlighted through their significant reduction in ΔP_a_CO_2_ between ΔD2/3 and ΔD9/10 (*p* < 0.0001, Figure 4D). This differs from females as P_a_CO_2_ is reduced with ascent but is not significantly different between ΔD2/3 and ΔD9/10 (*p* = 0.8632, Figure 4D). This more gradual decline in P_a_CO_2_ may delay the stimulus for HCO_3_^−^ excretion by the kidneys, thus prolonging the time needed for renal compensation to correct the alkalosis fully. Male renal tubules may be optimized for acid excretion and bicarbonate conservation, reflecting their evolutionary adaptation to higher muscle mass and therefore greater acid loads (Harris et al. 2019, 2020). While beneficial in conditions of acidemia, these traits may slow HCO_3_^−^ excretion during alkalosis. The combination of higher baseline [HCO_3_^−^]_a_ levels and a slower ventilatory response, resulting in a potential delay in activation of renal bicarbonate excretion, all likely contribute to the prolonged time course of renal compensation in males during HA exposure.

At 1100 m, P_a_CO_2_ and [HCO_3_^−^]_a_ were significantly lower in females, and thus, one may expect pH_a_ to be significantly lower in females. However, pH_a_ was not significantly different between males and females at 1100 m. This result was also consistent within the expanded data set from our laboratory (*n* = 80; 34 F, 46 M; Figure 6) and previous studies (Crapo et al. 1999; Klæstrup et al. 2011). Similar pH_a_ between sexes, despite differing P_a_CO_2_ and [HCO_3_^−^]_a_ is most likely due to baseline renal compensation in females and additional buffering from blood proteins. M. P. Fitzgerald (1913, 1914), noted that females exhibit a more responsive renal compensation at baseline, due to increased ventilation, potentially contributing to stable pH_a_ despite lower P_a_CO_2_ and [HCO_3_^−^]_a_. While the bicarbonate buffer system is the major blood buffer system, there are others, such as plasma protein albumin (e.g., Ellison et al. 1958) and hemoglobin (e.g., Antonini et al. 1967). Proteins are composed of amino acids containing carboxyl and amino groups and side chains that can act as weak acids or bases (e.g., Ellison et al. 1958; Kochansky et al. 2008). These groups can either donate protons when pH rises (alkalemia) or accept protons when pH falls (acidemia), thus stabilizing pH_a_ (e.g., Kochansky et al. 2008). Albumin is the most abundant plasma protein and contributes to buffering via its histidine residues, which have an imidazole side chain that can accept or donate H^+^ depending on blood pH (e.g., Rothschild et al. 1988). Proteins work with other buffering systems (e.g., bicarbonate) to help modulate pH_a_. However, as P_a_CO_2_ and [HCO_3_^−^]_a_ are proportional within the sexes, the observed balance in pH_a_ at 1100 m further suggests that chemistry‐based equilibrium plays a role in buffering sex differences, rather than proteins alone. Therefore, blood proteins as well as chemistry‐based equilibrium play a role in mitigating pH changes, thereby contributing to blood pH maintenance.

### 4.7 Methodological Considerations

Arterial blood draws via an indwelling catheter are the gold standard for blood gas, electrolyte, and acid–base sampling and measurement, including P_a_CO_2_, [HCO_3_^−^]_a_, pH_a_, [H^+^]_a_, as well as P_a_O_2_ and S_a_O_2_ (e.g., Dar et al. 1995; Pandit 1995; Zouboules et al. 2018; Bird et al. 2021). Arterial blood draws require medical expertise (e.g., trained physician or respiratory therapist), and single arterial draws (e.g., radial artery) are associated with risks including pain (e.g., Barry et al. 1995) and transient hyperventilation, which has the potential to cofound resting P_a_CO_2_ and thus, acid–base variables. However, P_a_CO_2_ values in the present study was similar to those reported in previous arterial blood draws upon rapid ascent and residence to high altitude using single arterial blood draws (Zouboules et al. 2018; Bird et al. 2021), and similar in comparison to baseline values using indwelling radial arterial catheters (Russell et al. 2024).

We utilized a portable blood gas and electrolyte analyzer (Abbott i‐STAT1) in this field‐based study, which offered convenient cartridge‐based analysis of arterial blood samples. The i‐STAT CG4+ cartridges measure PCO_2_ and pH directly via a miniaturized Severinghaus electrode and potentiometric glass electrode, respectively, similar to the traditional benchtop blood gas analyzers (e.g., Radiometer). [HCO_3_^−^] is subsequently‐calculated from PCO_2_ and pH using the Henderson–Hasselbach equation in all blood gas analysis systems. Thus, the acid–base metrics we report here are likely a true representation of participant acid–base status relative to gold standard research and clinical devices.

However, the Abbott i‐STAT CHEM8+ cartridges utilize differential technology for PO_2_ and SO_2_ measures. Although PO_2_ is measured via miniature amperometric Clarke‐style electrode similar to a traditional benchtop analyzer, SO_2_ and [Hb] are not measured directly via combined oximetry (CO‐OX) using spectrophotometry. Rather, SO_2_ is calculated from PO_2_ and pH utilizing the oxy‐hemoglobin dissociation curve, and [Hb] is calculated from hct, which is measured via conductivity. Although PaO_2_ and SaO_2_ were decreased upon immediate ascent (D2/3) as expected, surprisingly these variables were not increased with ventilatory acclimatization on D9/10 (illustrated by reductions in PaCO_2_ and calculated alveolar ventilation). Specifically, we noted that PaO_2_ and SaO_2_ were not increased over time at 3500 m in the larger participant pool (Table 1 and Figure 1), nor when stratified by sex (Table 2 and Figure 3). However, [Hb] was increased with residence at 3500 m (likely due to plasma volume contraction; e.g., Bouten et al. 2026; Tremblay et al. 2025), which translated to increases in CaO_2_. Given (a) the differences in technology regarding the measurement of oxygenation between the portable i‐STAT system and more traditional benchtop analyzers, and (b) the data we report here, we conclude that measures of oxygenation should be interpreted with caution when utilizing portable cartridge‐based systems without co‐oximetry with ascent and residence at high altitude, where PO_2_ values are lower than sea level (e.g., Gunsolus et al. 2017).

While we did not control for menstrual cycle phase or exogenous hormonal contraceptive use, due to the logistical constraints of our pre‐ and post‐ascent testing schedule, these factors are unlikely to confound our findings. Pre‐menopausal females consistently exhibit higher steady‐state resting minute ventilation during the mid‐to‐late luteal phase compared to the follicular phase (e.g., MacNutt et al. 2012). Elevated circulating progesterone acts centrally to lower the medullary central chemoreceptor set‐point for carbon dioxide (Pascual et al. 2002), driving increases in resting baseline ventilation and concomitant mild hypocapnia at both sea level and altitude, a physiological shift similar to hyper‐progesterone states like pregnancy (e.g., Jensen et al. 2007). Furthermore, females maintain higher ventilation relative to males regardless of cycle phase (Loeppky et al. 2001), indicating that this overall sex difference in baseline ventilatory drive persists above normal hormonal fluctuations and other background variations such as diet, sleep, and physical activity.

While baseline ventilatory drive fluctuates cyclically in pre‐menopausal females, HVR gain remains stable across the menstrual cycle. Although early research suggested that luteal‐phase hypocapnia suggests heightened hypoxic sensitivity (Takano 1984), newer methodologically rigorous studies controlling for carbon dioxide demonstrate that acute HVR does not meaningfully change across the ovarian cycle. Specifically, isocapnic HVR and hypoxic exercise ventilation show no significant differences across follicular, late‐follicular, and mid‐luteal phases at rest or during exercise (MacNutt et al. 2012). Menstrual phase variations also do not alter peripheral oxygen saturation during acute nitrogen breathing, susceptibility to acute mountain sickness, or overall high‐altitude tolerance (Richalet et al. 2020; Citherlet et al. 2024), with endogenous hormonal shifts falling well within normal day‐to‐day inter‐individual variability in chemosensitivity (Oeung et al. 2023; Grittner et al. 2025).

A clear physiological contrast emerges between the sexes, wherein pre‐menopausal females exhibit higher steady‐state central ventilatory drive than males across their cycle, but males demonstrate higher transient peripheral HVR gain. Males exhibit greater HVR gain at sea level (Aitken, Franklin, and Cameron 1986; Aitken, Franklin, Pierson, and Schoene 1986; Raberin et al. 2023), during acute hypoxia (MacKenzie et al. 2026; Oliveira et al. 2025), and throughout high‐altitude acclimatization (Schoene et al. 1990; Muza et al. 2001; Lhuissier et al. 2012). This higher peripheral loop gain in males directly contributes to their higher incidence of central sleep apnea (CSA) upon ascent (Bird et al. 2023). Because the menstrual cycle primarily alters baseline central drive rather than acute peripheral HVR gain, exact cycle timing is less critical when interpreting the overarching sex differences demonstrated in this novel context. Nevertheless, future studies investigating the effects of sex on blood oxygenation and acid–base acclimatization should account for ovarian cycle phases and hormonal contraceptive use, while explicitly differentiating between steady‐state central drive and acute HVR responsiveness.

### 4.8 Potential Clinical Relevance

The findings of this study may be clinically‐relevant in understanding sex‐specific differences in renal physiology and their potential implications for kidney disease, hypertension, metabolic acidosis, and sleep‐disordered breathing, particularly CSA at HA or in the context of heart failure. The observation that females exhibit faster and more robust renal compensation to respiratory alkalosis, likely due to transporter expression and hormonal regulation differences, underscores the potential for sex‐specific vulnerability and responses to renal pathologies. For instance, the reduced baseline [HCO_3_^−^] and enhanced bicarbonate excretion in females with early ascent may predispose them to distinct acid–base imbalances, such as a greater susceptibility to metabolic acidosis in conditions that demand sustained acid–base buffering (Raphael et al. 2016). Conversely, the male tendency for slower renal compensation and greater bicarbonate retention could contribute to a heightened risk of developing chronic conditions like hypertension, as increased sodium and bicarbonate retention are known to influence blood pressure regulation (e.g., Luft et al. 1990). Additionally, the slower renal compensation and higher [HCO_3_^−^] levels in males may exacerbate CSA at HA, a condition associated with repetitive oscillations in respiratory pattern due in part to increases in transient chemoreflex loop gain (Bird et al. 2023; Lombardi et al. 2013; Caravita et al. 2015). The physiological predisposition illustrated here may explain increased susceptibility to CSA, as males experience an increased degree of sleep disordered breathing compared to females at HA (e.g., Bird et al. 2023). These findings could inform personalized approaches to managing respiratory and renal diseases, acid–base disorders, and altitude‐related conditions. By recognizing these physiological differences, clinicians could tailor therapeutic interventions to optimize outcomes based on patient sex and underlying renal function. These insights contribute to a deeper understanding of the interplay between respiratory and renal physiology, and highlight the need for sex‐specific approaches in diagnosing and treating renal and altitude‐related disorders.

## 5 Conclusions

We evaluated the integrative ventilatory and renal acid–base acclimatization differences between females and males following rapid ascent and a 10‐day residence at 3500 m. We found that at baseline (D0, 1100 m), females exhibited significantly lower P_a_CO_2_ and [HCO_3_^−^]_a_ than males, suggesting a higher ventilatory drive and enhanced bicarbonate excretion in females. At early high altitude (E‐HA; D2/3), females had significantly higher ventilation ($\dot{V}$_A_) and P_a_O_2_, and significantly lower P_a_CO_2_, [HCO_3_^−^]_a_, and pH_a_ compared to males. However, no significant differences in ${\dot{V}}_{A}$, P_a_CO_2_, [HCO_3_^−^]_a_, and pH_a_ were observed between males and females by late high altitude (L‐HA; D9/10). This indicates that males require a longer time course to achieve the same level of compensatory renal response as females. These findings highlight novel sex‐based differences in the time course and magnitude of ventilatory and renal acclimatization mechanisms, with females demonstrating a more robust ventilatory and rapid renal response upon rapid ascent to HA. We propose that this more rapid and robust response to HA in females is due in part to their baseline lower P_a_CO_2_ and [HCO_3_^−^]_a_, which better predisposes them to the stressors of HA. To our knowledge, this study is the first to directly‐compare ventilatory and renal acid–base acclimatization between sexes during sustained residence at 3500 m, providing critical insights into the physiological mechanisms underlying sex‐specific acclimatization patterns. This work emphasizes the importance of considering sex differences in future studies of high‐altitude acclimatization and in the management of altitude‐related disorders.

## Author Contributions

R.J.A.W. and T.A.D., conception, design and planning of this work. B.W.L.M., N.A.J., R.I., J.L.K., G.Z.‐C., N.D.J.S., R.J.A.W. and T.A.D., organization, protocol and data collection; N.V.B., B.W.L.M., N.A.J., N.D.J.S., R.J.A.W. and T.A.D., data analysis and interpretation; All authors critically evaluated the work, edited and approved the final manuscript draft. All authors listed are qualified to be co‐authors, and all those qualified to be co‐authors are listed.

## Funding

This study was funded in part by the Natural Sciences and Research Council of Canada (NSERC) Undergraduate Student Research Awards (N.V.B., N.A.J.), Alberta Innovates Summer Studentship (B.W.L.M.), NSERC Discovery Grants (T.A.D., RGPIN‐2024‐04182; R.J.A.W., RGPIN‐2020‐05312), Canadian Institutes of Health Research Project Grant (R.J.A.W., RN291964‐366421), University of Calgary Transdisciplinary Connector Grant (T.A.D., R.J.A.W.), and the Hotchkiss Brain Institute—Rebecca Hotchkiss International Scholar Exchange (HBI‐RHISE) program (R.J.A.W.).

## Consent

Informed verbal and written consent was obtained in advance from all individual participants included in the study. The authors affirm that human research participants provided informed consent for publication of the deidentified, numerical data.

## Conflicts of Interest

The authors declare no conflicts of interest.

## Data Availability

The de‐identified, numerical data from this study can be provided to any qualified researcher upon reasonable request to the senior author.
